# Assembling spatial clustering framework for heterogeneous spatial transcriptomics data with GRAPHDeep

**DOI:** 10.1093/bioinformatics/btae023

**Published:** 2024-01-18

**Authors:** Teng Liu, Zhaoyu Fang, Xin Li, Lining Zhang, Dong-Sheng Cao, Min Li, Mingzhu Yin

**Affiliations:** Department of Clinical Research Center (CRC), Clinical Pathology Center (CPC), Cancer Early Detection and Treatment Center (CEDTC) and Translational Medicine Research Center (TMRC), Chongqing University Three Gorges Hospital, Chongqing University, Wanzhou, Chongqing, 404000, China; Department of Chongqing Technical Innovation Center for Quality Evaluation and Identification of Authentic Medicinal Herbs, Chongqing, 400044, China; Department of Hunan Provincial Key Lab on Bioinformatics, School of Computer Science and Engineering at Central South University, Hunan, 410083, China; Department of Clinical Research Center (CRC), Clinical Pathology Center (CPC), Cancer Early Detection and Treatment Center (CEDTC) and Translational Medicine Research Center (TMRC), Chongqing University Three Gorges Hospital, Chongqing University, Wanzhou, Chongqing, 404000, China; Department of Chongqing Technical Innovation Center for Quality Evaluation and Identification of Authentic Medicinal Herbs, Chongqing, 400044, China; Department of Clinical Research Center (CRC), Clinical Pathology Center (CPC), Cancer Early Detection and Treatment Center (CEDTC) and Translational Medicine Research Center (TMRC), Chongqing University Three Gorges Hospital, Chongqing University, Wanzhou, Chongqing, 404000, China; Department of Chongqing Technical Innovation Center for Quality Evaluation and Identification of Authentic Medicinal Herbs, Chongqing, 400044, China; Department of Xiangya School of Pharmaceutical Sciences, Central South University, Changsha 410003, P.R. China; Department of Hunan Provincial Key Lab on Bioinformatics, School of Computer Science and Engineering at Central South University, Hunan, 410083, China; Department of Clinical Research Center (CRC), Clinical Pathology Center (CPC), Cancer Early Detection and Treatment Center (CEDTC) and Translational Medicine Research Center (TMRC), Chongqing University Three Gorges Hospital, Chongqing University, Wanzhou, Chongqing, 404000, China; Department of Chongqing Technical Innovation Center for Quality Evaluation and Identification of Authentic Medicinal Herbs, Chongqing, 400044, China

## Abstract

**Motivation:**

Spatial clustering is essential and challenging for spatial transcriptomics’ data analysis to unravel tissue microenvironment and biological function. Graph neural networks are promising to address gene expression profiles and spatial location information in spatial transcriptomics to generate latent representations. However, choosing an appropriate graph deep learning module and graph neural network necessitates further exploration and investigation.

**Results:**

In this article, we present GRAPHDeep to assemble a spatial clustering framework for heterogeneous spatial transcriptomics data. Through integrating 2 graph deep learning modules and 20 graph neural networks, the most appropriate combination is decided for each dataset. The constructed spatial clustering method is compared with state-of-the-art algorithms to demonstrate its effectiveness and superiority. The significant new findings include: (i) the number of genes or proteins of spatial omics data is quite crucial in spatial clustering algorithms; (ii) the variational graph autoencoder is more suitable for spatial clustering tasks than deep graph infomax module; (iii) UniMP, SAGE, SuperGAT, GATv2, GCN, and TAG are the recommended graph neural networks for spatial clustering tasks; and (iv) the used graph neural network in the existent spatial clustering frameworks is not the best candidate. This study could be regarded as desirable guidance for choosing an appropriate graph neural network for spatial clustering.

**Availability and implementation:**

The source code of GRAPHDeep is available at https://github.com/narutoten520/GRAPHDeep. The studied spatial omics data are available at https://zenodo.org/record/8141084.

## Graphical abstract

**Graphical abstract:**
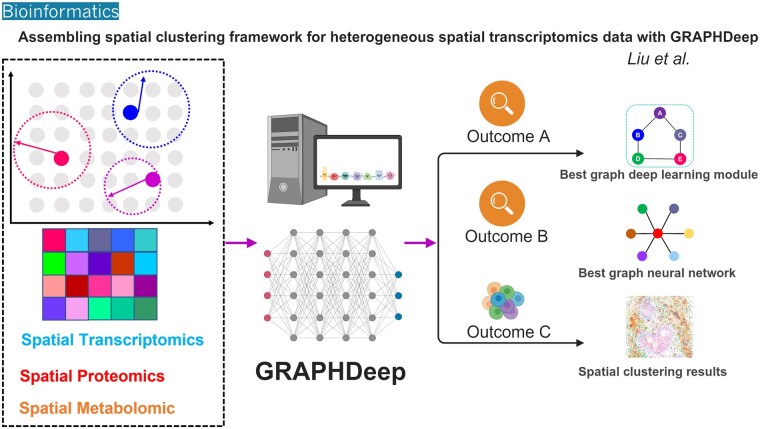

## 1 Introduction

Spatial omics techniques enable biomolecular analysis by preserving the spatial context of cells within the tissue microenvironment (Berrios *et al.* 2021). These technologies encompass spatial transcriptomics, epigenomics, proteomics, and metabolomics. Specifically, spatial transcriptomics includes platforms, such as 10X Visium (Prakrithi *et al.* 2023), 10X Xenium (Salas *et al.* 2023), seqFISH+ (Eng *et al.* 2019), and Slide-seqV2 (Stickels *et al.* 2021). Spatial proteomics techniques encompass 4i (Gut *et al.* 2018), MIBI-TOF (Keren *et al.* 2019), and IMC (Jackson *et al.* 2020). Spatial metabolomics integrates SEAM (Yuan *et al.* 2021) and SpaceM (Rappez *et al.* 2021). These high-throughput sequencing data generated across diverse methodologies exhibit distinctive modalities, resolutions, and scales.

Spatial clustering constitutes a foundational and indispensable procedure within spatial omics research (Zeng *et al.* 2022). It profoundly influences downstream analyses’ accuracy and performance, spanning spatial deconvolution, gene imputation, spatial organization reconstruction, cell–cell communication, and spatial trajectory inference. Diverse methodologies have emerged to address spatial clustering. For instance, Louvain, KMeans, and Leiden (Traag *et al.* 2019) are traditional approaches for handling gene expression features *in situ* sequencing data. Bayesian statistical models underlie BayesSpace (Zhao *et al.* 2021) and SC-MEB (Yang *et al.* 2022) to integrate spatial coordinates and gene expression. Leveraging morphological similarity and neighborhood smoothing, stLearn (Pham *et al.* 2020) segregates clusters within morphological images. By aggregating contiguous pixels within digital images, MULTILAYER (Moehlin *et al.* 2021) elucidates biological substructures.

Recent advancements in graph neural networks (GNNs) have unveiled unprecedented potential for integrating spatial position and gene expression information, facilitating spatial clustering tasks. GNNs, particularly when embedded within robust graph deep learning (GDL) modules, hold promise for discriminating spatial groups. Notably, SpaGCN (Hu *et al.* 2021) employs the traditional graph convolutional network (GCN) to capture cluster heterogeneity, integrating spatial location, gene expression, and histology. SEDR (Fu *et al.* 2021) and STAGATE (Dong and Zhang 2022) utilize graph autoencoders for managing spatial location profiles, wherein derived latent embeddings enhance clustering accuracy. CCST (Li *et al.* 2022) extends the unsupervised deep graph infomax (DGI) module to maximize mutual information between global summaries and local representations, creating latent embeddings. Furthermore, DeepST (Xu *et al.* 2022) utilizes variational graph autoencoders (VGAE) and denoising autoencoders for processing features from spatial transcriptomes and generating latent representations. However, existing spatial clustering architectures lack compatibility with heterogeneous spatial omics data. Clustering accuracy is influenced by sequencing quality and spatial data resolution. These frameworks lack adequate justification for selecting specific GDL module and GNN. Moreover, extensibility in modules, GNNs, and data diversity remains insufficient.

To address these limitations, this manuscript introduces GRAPHDeep, a novel approach for accomplishing spatial clustering tasks by employing an array of graph learning modules and neural networks. GRAPHDeep integrates two robust GDL modules, VGAE (Kipf and Welling 2016) and DGI (Veličković *et al.* 2018), utilizing 20 GNNs as encoders and decoders. This encompasses a total of 40 distinct GNN-based frameworks, each contributing to the spatial clustering objective. These frameworks are applied across various spatial omics data types, including 10X Visium (Prakrithi *et al.* 2023), Slide-seqV2 (Stickels *et al.* 2021), 4i (Gut *et al.* 2018), MERFISH (Lu *et al.* 2021), among others, to generate latent representations within an embedding space. Consequently, a versatile GDL architecture for spatial clustering is assembled, catering to diverse data types. Furthermore, comparative experiments are conducted against state-of-the-art methods [STAGATE (Dong and Zhang 2022), spaGCN (Hu *et al.* 2021), DeepST (Xu *et al.* 2022), CCST (Li *et al.* 2022), SEDR (Fu *et al.* 2021), stLearn (Pham *et al.* 2020), BayesSpace (Zhao *et al.* 2021), and Scanpy (Wolf *et al.* 2018)] to demonstrate the efficacy of the GNN-based clustering methodologies. The goal of GRAPHDeep is to showcase that GNN-based clustering methods can achieve improved performance by selecting the right GNN. GRAPHDeep exhibits remarkable extensibility across the GDL module, GNNs, and spatial omics data, rendering it highly versatile for comprehensive analyses of spatially resolved transcriptomes.

## 2 Materials and methods

This section outlines the operational framework of GRAPHDeep, encompassing the overarching architecture (Section 2.1), VGAE and DGI modules, spatial omics data, and benchmarking methods. The VGAE and DGI modules are implemented in Python using PyTorch_pyG (Fey and Lenssen 2019). Both modules are unsupervised learning models (Section 2.2), rendering labeled data unnecessary for module training. The spatial omics data include 10X Visium, 4i, Slide-seqV2, and MERFISH (Section 2.3). Benchmarking methods used encompass STAGATE, spaGCN, DeepST, CCST, SEDR, stLearn, BayesSpace, and Scanpy (Section 2.4).

### 2.1 Overall architecture of GRAPHDeep

**Figure 1.**
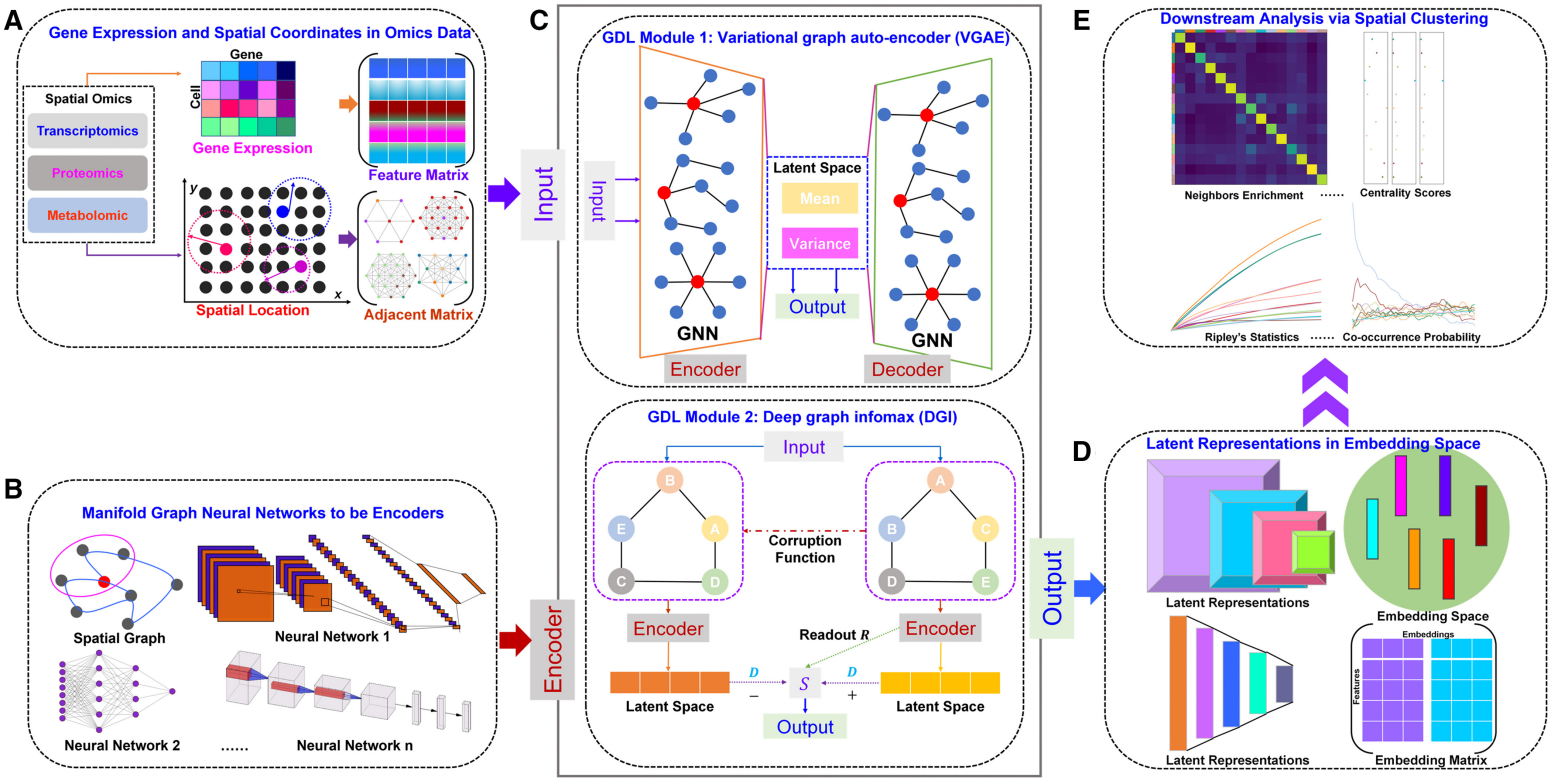
The schematic architecture of GRAPHDeep. (A) Practicable spatial omics data in GRAPHDeep. The data are processed as the feature matrix and adjacency matrix to import the GDL module. (B) A crowd of GNNs. In the VGAE module, these GNNs are used as encoders and decoders to mimic the similarity between two graphs. In the DGI module, these GNNs are taken as encoders to represent the positive and negative samples. (C) Two GDL modules, VGAE, and DGI. These modules could enhance the learning ability of a single GNN. (D) The embedding space of latent representations. The generated latent embeddings are applied to segment the spatial domains. (E) The feasible downstream analysis of each spatial cluster. These analytical parameters could reflect each method’s clustering accuracy and declare the biological meaning of spatial domains.

Figure 1 illustrates the comprehensive schematic diagram of GRAPHDeep. The core segment encompasses the GDL module (Fig. 1C). This study examines and contrasts two pivotal modules, VGAE and DGI. Surrounding the GDL modules, three distinct constituents emerge: input, output, and encoder. The spatial omics data serve as GRAPHDeep’s input (Fig. 1A). Specifically, four spatial datasets are analyzed for spatial clustering. Each individual entity within spatial omics data is interpreted as a cell, pixel, or spot (referred to as “spot” for brevity). The feature matrix is formulated based on gene expression profiles, while the adjacent matrix is derived from the spatial location information (Supplementary Fig. S1).

In GRAPHDeep, the encoders comprise diverse GNNs (Fig. 1B). Given the current literature’s lack of consensus on GNN selection, GRAPHDeep assesses 20 GNNs (Supplementary Table S1) within two GDL modules. These GNNs encompass prominent convolutional neural networks like auto-regressive moving average (ARMA), graph attention network (GAT), hypergraph convolution, molecular fingerprint (MF), self-supervised GAT, topology adaptive graph (TAG), among others (Wu *et al.* 2021) (Supplementary Table S1). To integrate GNNs and spatial omics data, the optimal GNN for each dataset is identified. These findings inform GNN selection in the dynamic landscape of spatial clustering research.

Post-training, spatial features yield latent representations in the embedding space (Fig. 1D). Quality of latent embeddings significantly influences segmentation accuracy and performance. Subsequent to clustering, downstream analysis would be conducted (Fig. 1E). For instance, co-occurrence probability between two clusters may elucidate tissue development and disease progression. Neighbor enrichment scores across groups shed light on tumor infiltration and gene–gene interaction. We identify superior GNNs for spatial clustering based on performance across datasets and calculate average rankings. Comprehensive exposition of GRAPHDeep’s capabilities is detailed in the subsequent experimental results.

### 2.2 GDL modules

#### 2.2.1 Deep graph infomax

As shown in Fig. 1A, the DGI module (Veličković *et al.* 2018) takes the feature matrix X and adjacent matrix A as inputs. In specific spatial omics data, the feature matrix arises from normalized variance of highly variable genes. The adjacent matrix depends on spot-to-spot distances, computed via the radius neighbor model or *k*-nearest neighbor model (Wang *et al.* 2020).

The DGI module (Fig. 1C) comprises four essential elements: the corruption function C, encoder E, readout function R, and discriminator D. Initially, the corruption function generates negative samples as $\left(\bar{X},\bar{A})=C(X,A\right)$ and is achieved by the *torch.randperm* function in Pytorch. Subsequently, the encoder transforms the feature and adjacent matrices to yield latent embeddings:

(1) $$\begin{array}{c} h=\;E\left(X,A\right)\\ \bar{h}=\;E\left(\bar{X},\bar{A}\right), \end{array}$$

where encoder implies the chosen GNN. *h* and $\bar{h}$ are the latent embeddings with respect to the positive and negative samples. The readout function is further employed to create the global summaries:

(2) $$S=R\left(E\left(X,A\right)\right).$$

Finally, the mutual information between the local representations and global summaries is signified by the discriminator as a probability score D(h,S) and $D(\bar{h},S)$. Without a doubt, the training objective is associated with these two mutual information. Following the original DGI module, a standard binary cross-entropy loss function (Veličković *et al.* 2018) is formulated:

(3) $$L_{D}=E_{\left(X,A\right)}\left[\log D\left(h,S\right)\right]+E_{\left(\bar{X},\bar{A}\right)}\left[\log(1-D(\bar{h},S))\right].$$

#### 2.2.2 Variational graph autoencoder

The critical reformation in VGAE is applying a two-layer GNN structure to generate the latent embedding. The first-layer GNN decides a lower-dimensional feature matrix $\tilde{X}$ as (Kipf and Welling 2016):

(4) $$\begin{array}{c} \tilde{X}=\mathrm{GNN}\left(X,A\right)=\mathrm{ReLU}(\tilde{A}XW_{0})\\ \tilde{A}=D^{-\frac{1}{2}}AD^{-\frac{1}{2}}, \end{array}$$

where $\tilde{A}$ is the symmetrically normalized adjacency matrix. Re⁡LU(.)=max⁡(0,.) is the rectified linear activation function. $W_{0}$ is the weight parameter for this layer GNN. Here, GNN has the sames meaning as encoder.

In the second-layer GNN, the mean and variance vectors (Fig. 1C) of feature matrix are computed with weight parameters $W_{1}$:

(5) $$\begin{array}{c} \mu ={\mathrm{GNN}}_{\mu }\left(X,A\right)=\tilde{A}\tilde{X}W_{1}\\ l\mathrm{og}\sigma^{2}={\mathrm{GNN}}_{\sigma }\left(X,A\right)=\tilde{A}\tilde{X}W_{1}. \end{array}$$

These two values share the same weight parameters. Then, the latent representations are deduced via the reparameterization trick (Kingma and Welling 2014):

(6) Z=μ+σ⊙ϵ,

where ϵ∈Norm(0,1) is the standard normal distribution. In the VGAE module, the incoming and predictive graphs are expressed with probability distributions, i.e. q(Z|X,A) and p(A|Z). The adjacency matrix is reconstructed by decoder (inner product) as:

(7) $$p\left(A|Z\right)=\sigma (Z\;{\cdot \;Z}^{T}).$$

The loss function encompasses two error categories. The initial component quantifies reconstruction error, assessing input–output graph resemblance. The second minimizes discrepancies between distributions *q* and *p*. This loss is expressed as follows:

(8) $$L_{V}=E_{q\left(Z|X,A\right)}\left[\log p\left(A|Z\right)\right]-KL(q(Z|X,A))||p\left(Z\right),$$

where *KL*(.) indicates Kullback–Leibler divergence between two probability distributions.

This study assesses the encoder in DGI and VGAE modules across 20 GNNs (Supplementary Table S1), utilizing Python’s PyTorch_pyG (Fey and Lenssen 2019). These recently published neural networks generate latent embeddings for spatial domain identification. Within benchmarking methods, one GNN is incorporated, potentially not being the optimal selection. The hardware configuration and hyperparameters in GNNs are described in Supplementary Note S1.1. For various spatial omics datasets with different genes, the hyperparameters remain unchanged for a fair comparison purpose.

### 2.3 Spatial omics data

Four spatial omics datasets are involved in this article. Human dorsolateral pre-frontal cortex (DLPFC) spatial transcriptomics data are accessible via the 10X Visium platform. The spatialLIBD project (Pardo *et al.* 2022) presents an extensive analysis, encompassing spot-level, layer-level data, and spatial marker genes. These data comprise 12 samples, each spanning multiple neuronal layers and white matter. Among these, eight samples exhibit seven clusters, while the remaining four samples manifest five groups. To assess GRAPHDeep, Slice 151673, containing 3639 spots and 33 538 genes, is utilized for evaluation, albeit without universal application.

Iterative indirect immuno-fluorescence imaging (4i) (Kramer *et al.* 2023) constitutes a high-throughput method for spatial protein detection. Leveraging immuno-fluorescence microscopy, over 40 heterogeneous proteins are discernible across varied spatial scales within biological samples. Employing 4i technology facilitates quantification of expression levels and captures subcellular distribution. The 4i approach enables quantification of protein abundance, phosphorylation, and sub-compartmentalization. Squidpy (Palla *et al.* 2022) houses the 4i dataset comprising 270 876 cells and 43 proteins. This study utilizes a subset of the 4i dataset, featuring 16 742 cells, 43 proteins, and 10 cell types, for spatial clustering experiments.

Slide-seqV2 (Stickels *et al.* 2021) stands as a near-cellular resolution spatial transcriptomics dataset, capturing mRNAs’ localization in mouse hippocampal neurons at 10 μm spatial resolution. This method thoroughly reproduces the initial Slide-seq protocol, encompassing library generation, bead synthesis, and array indexing. Focusing on GNN-based spatial clustering approaches, this article directly employs the Slide-seqV2 dataset from Squidpy. The pre-processed mouse hippocampus data, intended for spatial clustering study, comprise 26 146 cells, 4000 genes, and 14 groups.

MERFISH represents a massively multiplexed single-molecule imaging technology (Lu *et al.* 2021), capable of quantifying RNA species’ copy numbers and spatial positions within individual cells. The Vizgen data program includes multiple MERFISH datasets, notably the MERFISH mouse liver map and MERFISH mouse brain receptor map. This study concentrates on the pre-processed MERFISH mouse liver dataset from Squidpy (Palla *et al.* 2022). Similarly, a subset of the original data is extracted for spatial domain analysis to enhance computational efficiency. This subset encompasses 13 806 cells, 347 genes, and 28 groups.

### 2.4 Benchmarking methods

Benchmarked spatial clustering methods include SEDR, STAGATE, DeepST, spaGCN, CCST, stLearn, BayesSpace, and Scanpy. SEDR (Fu *et al.* 2021) introduces an unsupervised spatially embedded deep representation for spatial transcriptomics. It comprises two core components: gene expression representation via a deep autoencoder network and spatial information embedding through the VGAE module. STAGATE (Dong and Zhang 2022) integrates the GAT (Brody *et al.* 2021) into the fundamental autoencoder, addressing spatial coordinates and gene expression profiles. DeepST (Xu *et al.* 2022) employs two networks for spatially resolved transcriptomes. One is VGAE, integrating gene expression and spatial location; the other is a denoising autoencoder, producing latent representations. The distinctive GNN in spaGCN (Hu *et al.* 2021) is GCN, identifying spatial domains with coherent expression and histology.

CCST (Li *et al.* 2022) merges the DGI module and GCNs, deriving latent embeddings from a weighted hybrid adjacency matrix. stLearn (Pham *et al.* 2020) employs tissue morphology, gene expression, and spatial distance for spatial clustering. BayesSpace (Zhao *et al.* 2021) applies Bayesian statistics to integrate spatial coordinates and gene expression, but its performance on simulated data is unsatisfactory. Scanpy (Wolf *et al.* 2018) serves as a Python toolkit for single-cell gene expression data analysis, including preprocessing, visualization, and clustering protocols suitable for spatial omics data. Louvain is the standard clustering algorithm in Scanpy.

Benchmarking methods are implemented according to their specifications and default parameters for equitable comparison. Supplementary Note S1.1 details spatial omics data preprocessing, neural network hyperparameters, and hardware configuration. The adjusted Rand index (ARI) serves as the spatial clustering metric (Supplementary Note S1.2).

## 3 Results

### 3.1 Clustering performance on DLPFC data

**Figure 2.**
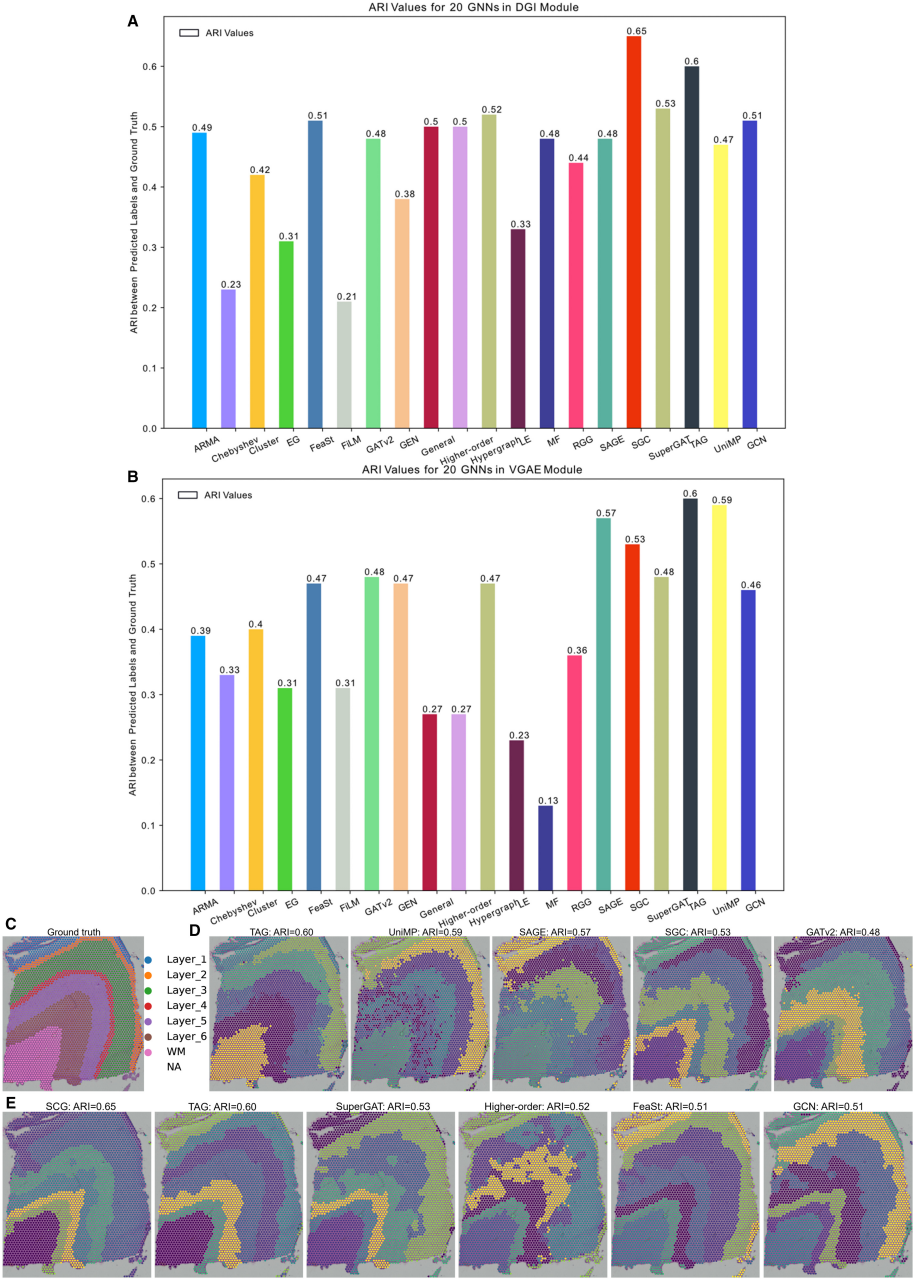
Experiment results of GRAPHDeep for the Sample 151673 in DLPFC data. (A) Clustering metric (ARI) for 20 GNNs with DGI module. The order of GNNs is the same as that in Supplementary Table S1. (B) ARI values for 20 GNNs with VGAE module. ARI is calculated according to the predicted labels and annotated ground truth. (C) The ground truth of Sample 151673, including 6 layers and white matter, 3639 spots, and 33 538 genes. (D) Top five GNNs in the VGAE module. These GNNs are TAG, UniMP, SAGE, SGC, and GATv2. The highest ARI is 0.60. (E) Top five GNNs in the DGI module. These GNNs are SGC, TAG, SuperGAT, Higher-order, FeaSt, and GCN. The ARI of FeaSt is identical to that of GCN. The largest ARI in the DGI module is 0.65 (SGC).

GRAPHDeep is employed to construct the GNN-based spatial clustering framework on DLPFC dataset. GRAPHDeep is executed across 12 samples, with Slice 151673 serving as an illustrative example. The ground truth, featuring seven clusters (six layers and white matter), is illustrated in Fig. 2C. ARI values for 20 GNNs in DGI and VGAE modules are presented in Fig. 2A and B, respectively. The highest ARI in the DGI module is 0.65 (SGC), and in the VGAE module, it is 0.60 (TAG). The optimal spatial clustering framework for this sample emerges as SGC in the DGI module. SGC and TAG are the top two accurate GNNs for the DGI module (Fig. 2A). Interestingly, the worst-performing GNNs differ between the two GDL modules (FiLM for DGI, 0.21; MF for VGAE, 0.13), suggesting that GDL influences GNN performance. The spatial clustering results of the top five GNNs analyzed on the tissue sample are compared with the ground truth, as depicted in Fig. 2D and E. Unfortunately, none of these clustering frameworks could accurately identify Layer_4 in the ground truth. The highest achieved clustering accuracy only reaches an ARI of ∼0.6, primarily due to the unsupervised nature of the task. It is worth acknowledging that SGC with the DGI module comes closest to matching the ground truth (Fig. 2E). Comparing GNNs with similar ARI levels reveals interesting insights. For instance, TAG demonstrates an ARI value of 0.60 in both the DGI and VGAE modules. However, upon closer examination of spot distributions (Fig. 2D and E), it becomes evident that the spot distribution in the DGI module appears to be more favorable than that in VGAE. This observation aligns with the conclusion that the DGI module is better suited for this specific sample. Even under the same GDL condition (FeaSt sharing an identical ARI of 0.51 with GCN in DGI module), the spot distributions exhibit significant variations.

**Figure 3.**
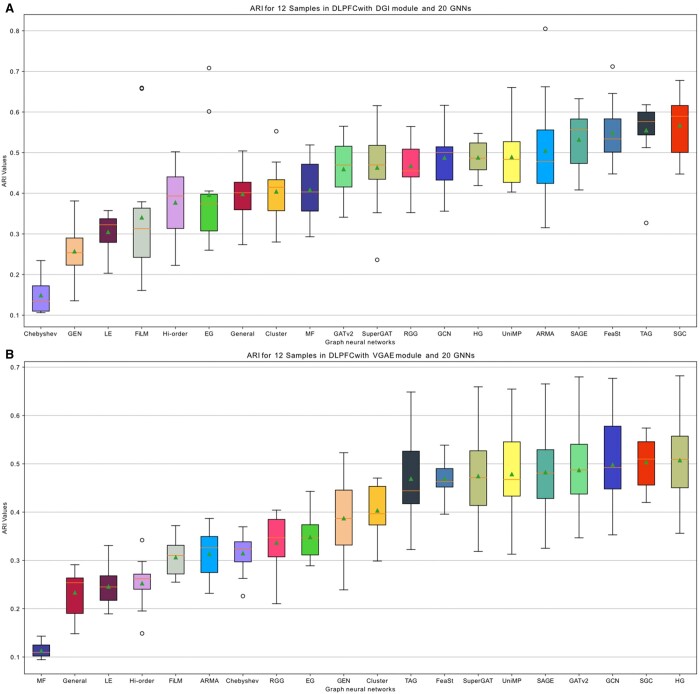
ARI values with respect to 20 GNNs in two DGL modules for 12 slices of DLPFC data. (A) ARI values of 20 GNNs in DGI module. The top five accurate GNNs are SGC, TAG, FeaSt, SAGE, and ARMA. Most of them are consistent with those for the individual Sample 151673. (B) The ARI values for 20 GNNs in VGAE module. The top five GNNs in this condition are Hypergraph, SGC, GCN, GATv2, and SAGE. Most of them are also in analogy with those for the single Slice 151673.

Figure 3A and B depicts ARI values using boxplots for 12 DLPFC data samples. The top five GNNs in these modules are SGC, TAG, FeaSt, SAGE, and ARMA, and Hypergraph, SGC, GCN, GATv2, and SAGE. In the DGI module, the top five ARI values surpass those in VGAE conditions, indicating DGI’s superior applicability to this DLPFC dataset. For this human DLPFC data, the recommended combination is DGI and SGC. Interestingly, the top five GNNs determined across 12 samples closely match those for the single 151 673 sample, such as SGC, TAG, FeaSt, GCN in the DGI module, and SGC, GATv2, SAGE, UniMP in the VGAE setting.

### 3.2 Computational efficiency evaluation

**Figure 4.**
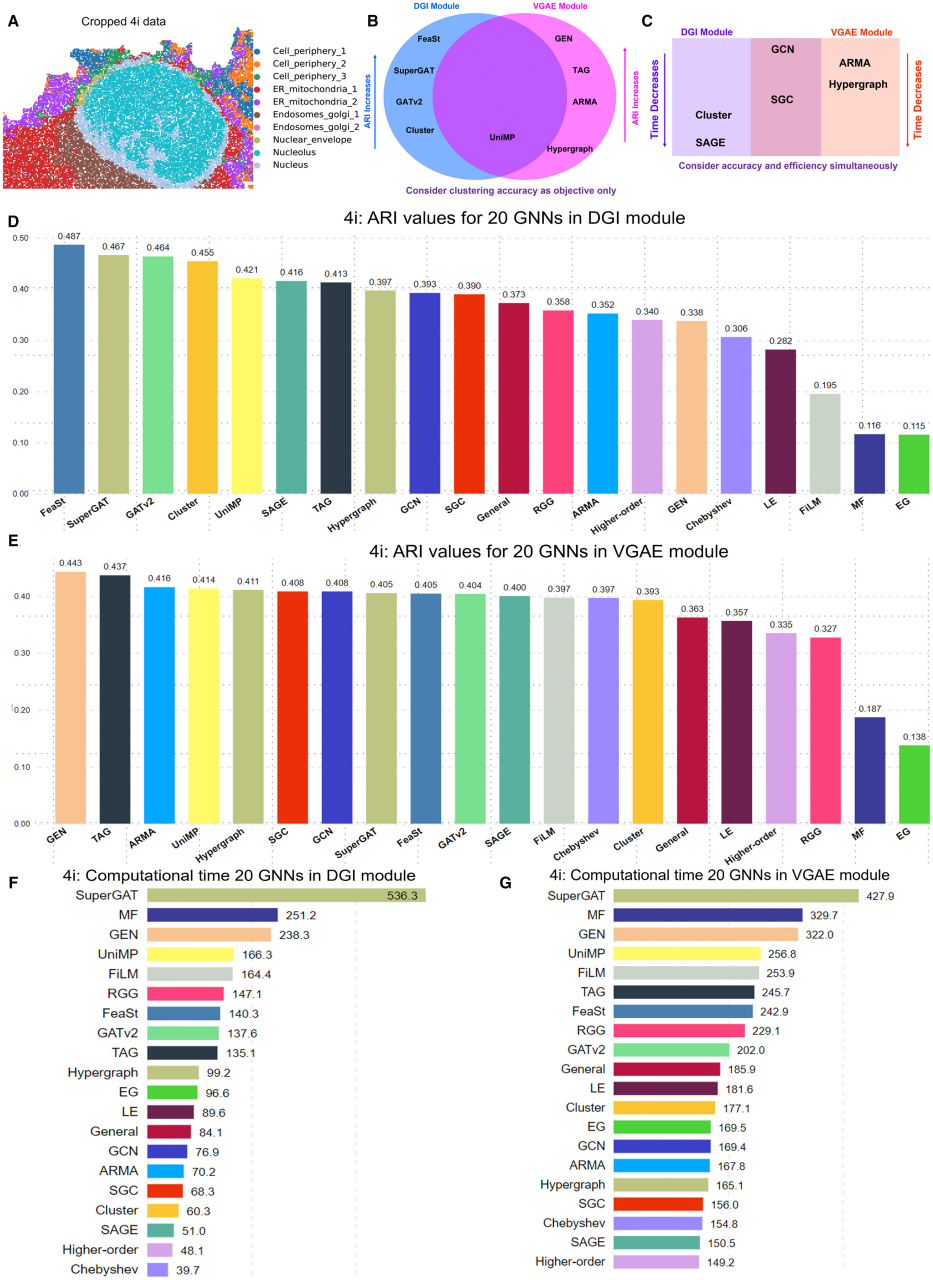
GRAPHDeep’s clustering results on 4i spatial protein data. (A) The subset 4i data contain 16 742 cells and 43 proteins in 10 cell types. Compared with the MIBI-TOF data (Supplementary Fig. S3), the number of cells, proteins, and groups increase simultaneously. (B) The top five GNNs in two modules are different and only has one overlap. (C) Trade-off GNNs between VGAE and DGI modules. These trade-off GNNs are selected from the top 10 accurate GNNs and top 10 efficient GNNs. (D) ARI values in the VGAE module. (E) ARI distribution in DGI modules. These ARI are larger than those in MIBI-TOF data (Supplementary Fig. S3E). The growth of proteins would result in higher ARI values. (F) Consumed time of each GNN in the VGAE module. (G) The computational time of 20 GNNs in the DGI module. For comparison, the elapsed time in 4i data is longer than that of MIBI-TOF data because of the promotion of cells and clusters.

GRAPHDeep is applied to two spatial protein datasets, 4i and MIBI-TOF, to establish an optimal spatial clustering structure and validate GNN candidate efficacy. Protein profiles inform the feature matrix derivation. Fewer proteins lead to an immature matrix and consequently inferior model performance, with calculative efficiency being a concern. The 4i subset comprises 16 742 cells/spots, 43 proteins, and 10 groups (Fig. 4A). Clusters are denser than MIBI-TOF data (Supplementary Fig. S3), with evident groups like ER_mitochondria_1, Endosomes_golgi_1, Nucleolus, and Nucleus. Additionally, 4i data boasts more proteins (43 versus 36 in MIBI-TOF), indicating potential for enhanced clustering performance.

Figure 4D and E presents ARI values of each GNN, with DGI slightly outperforming VGAE in this dataset. The spatial clustering results of top four GNNs in DGI module are depicted in Supplementary Fig. S2. The highest ARI in two modules are 0.49 and 0.44, respectively. Some GNNs share the same ARI values in this protein data, while MF and EG emerge as the least effective options. Despite the increased spot count in 4i data, ARI values surpass those in MIBI-TOF data (Supplementary Fig. S3D and E), credited to protein/gene enhancement. This underscores the significance of selected informative genes or proteins in spatial clustering tasks.

Furthermore, the increasing spots and genes exacerbate computational load. 4i consumes more time than MIBI-TOF (Fig. 4F and Supplementary Fig. S3F). The same top five time-consuming GNNs appear in both GDL modules: FiLM, UniMP, GEN, MF, and SuperGAT (Fig. 4F and G), aligning with good ARI performance. For instance, VGAE includes GEN, TAG, and UniMP (Fig. 4D), and DGI features SuperGAT, GATv2, and UniMP (Fig. 4E). Consequently, GRAPHDeep can select GNNs based on distinct goals. For clustering accuracy alone, top GNNs from Fig. 4D and E are chosen. When considering performance-efficiency trade-offs, GNN candidates are detailed in Fig. 4C. Merely four GNNs overlap between the top 10 accurate and efficient GNNs in each module. SGC and GCN are the further overlaps between modules (Fig. 4C). In conclusion, DGI module is advisable for these data, and GNN selection should align with research goals, be it clustering accuracy or computational efficiency.

### 3.3 Benchmarking methods comparison

This section compares GRAPHDeep with two types of benchmarking clustering methods. Scanpy, BayesSpace, and stLearn are non-GNN clustering techniques. Five GNN-based clustering approaches include STAGATE, DeepST, CCST, spaGCN, and SEDR. Slide-seqV2 is a high-resolution spatial transcriptomics technology and the relevant number of sequencing genes is 4000, and thus the top 3000 highly variable genes are screened to constitute the feature matrix (Supplementary Note S1.1). MERFISH is an image-based single-cell resolution technology and can interrogate a low number of genes than Slide-seqV2. Its related number of sequencing genes is 347 and all genes are used to construct feature matrix for this data. Since stLearn and spaGCN rely on morphological images, they are excluded from analysis in Slide-seqV2 and MERFISH datasets. Comparison between GRAPHDeep and six baselines (STAGATE, DeepST, CCST, Scanpy, BayesSpace, and SEDR) is performed on Slide-seqV2 and MERFISH, with a specific focus on Sample 151673 of the DLPFC dataset.

**Figure 5.**
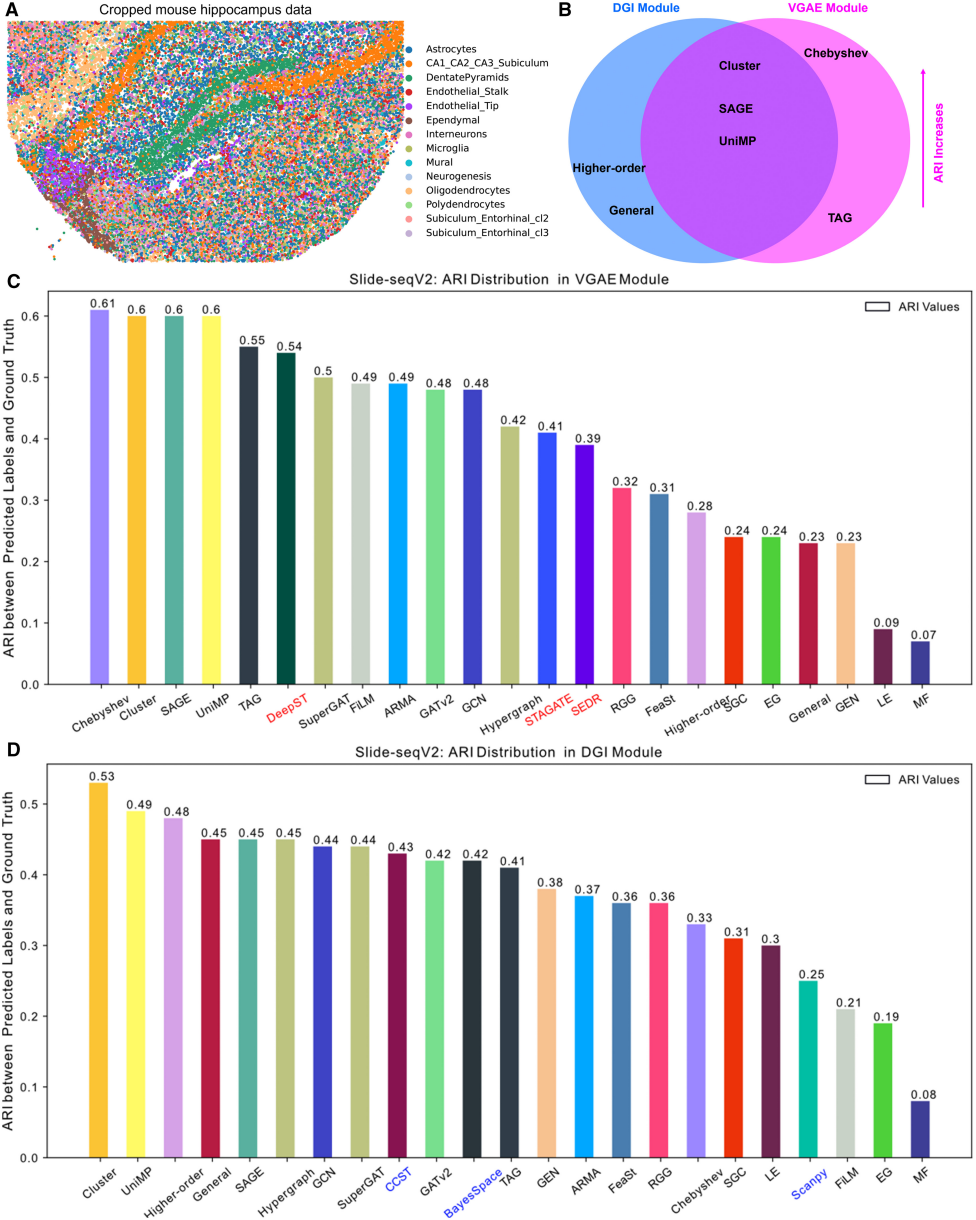
A comparison between GRAPHDeep and six benchmarking methods on Slide-seqV2-based mouse hippocampus data. (A) The subset of these data contains 26 146 cells, 4000 genes, and 14 groups. Slide-seqV2 has a near-cellular resolution. (B) Overlap top GNNs between DGI and VGAE modules. There are three intersections, Cluster, SAGE, and UniMP. (C) ARI values of 20 GNNs in the VGAE module. Three baseline methods (STAGATE, SEDR, and DeepST) are compared in the VGAE module. (D) ARI distribution of 20 GNNs in the DGI module. The VGAE module performs better than DGI.

Figure 5A depicts a segment of Slide-seqV2-based mouse hippocampus data, encompassing 26 146 cells, 4000 genes, and 14 clusters. Among these, only CA1_CA2_CA2_Subiculum and DentatePyramids groups show centralized distribution, while others exhibit dispersion. The ARI distributions for the two GDL modules are presented in Fig. 5C and D. VGAE is better than DGI module for this data and the spots’ distributions of top four GNNs in VGAE module are described in Supplementary Fig. S4. Figure 5C includes DeepST, STAGATE, and SEDR, while Fig. 5D involves CCST, BayesSpace, and Scanpy. STAGATE’s integration of a GAT yields accuracy akin to GATv2. SEDR’s performance (ARI = 0.39) closely aligns with STAGATE’s (ARI = 0.41). The best performance among the six benchmarks is seen in DeepST (ARI = 0.54), yet some GNNs in VGAE surpass DeepST. BayesSpace (ARI = 0.42) approximates CCST (ARI = 0.43), both surpassing Scanpy (Fig. 5D). In this dataset, VGAE outperforms the DGI module, achieving the highest ARI values of 0.61 and 0.53 for the two respective modules.

Figure 5B illustrates the intersectional GNNs in VGAE and DGI modules. Chebyshev demonstrates optimal precision with the shortest computation time (Supplementary Fig. S5A). Familiar GNN overlaps from previous experiments, Cluster, SAGE, and UniMP, are evident in Fig. 5B. Supplementary Figure S5A and B displays the elapsed times for the six benchmarking techniques. Similarly, DeepST’s duration surpasses STAGATE and SEDR (Supplementary Fig. S5A) due to its multiple neural networks. Comparatively, BayesSpace requires more time than CCST and Scanpy (Supplementary Fig. S5B). Notably, the top time-consuming GNNs are consistent across both modules, GEN, SuperGAT, MF, and FeaSt. The calculative time in DGI takes longer than VGAE. This observation suggests that GDL module choice would significantly affect computational time. For MERFISH data, we observe the similar results that GRAPHDeep outperforms the six benchmarking methods (Supplementary Fig. S6). Ultimately, the recommended module and GNN for both mouse hippocampus and MERFISH data are VGAE and Chebyshev.

**Figure 6.**
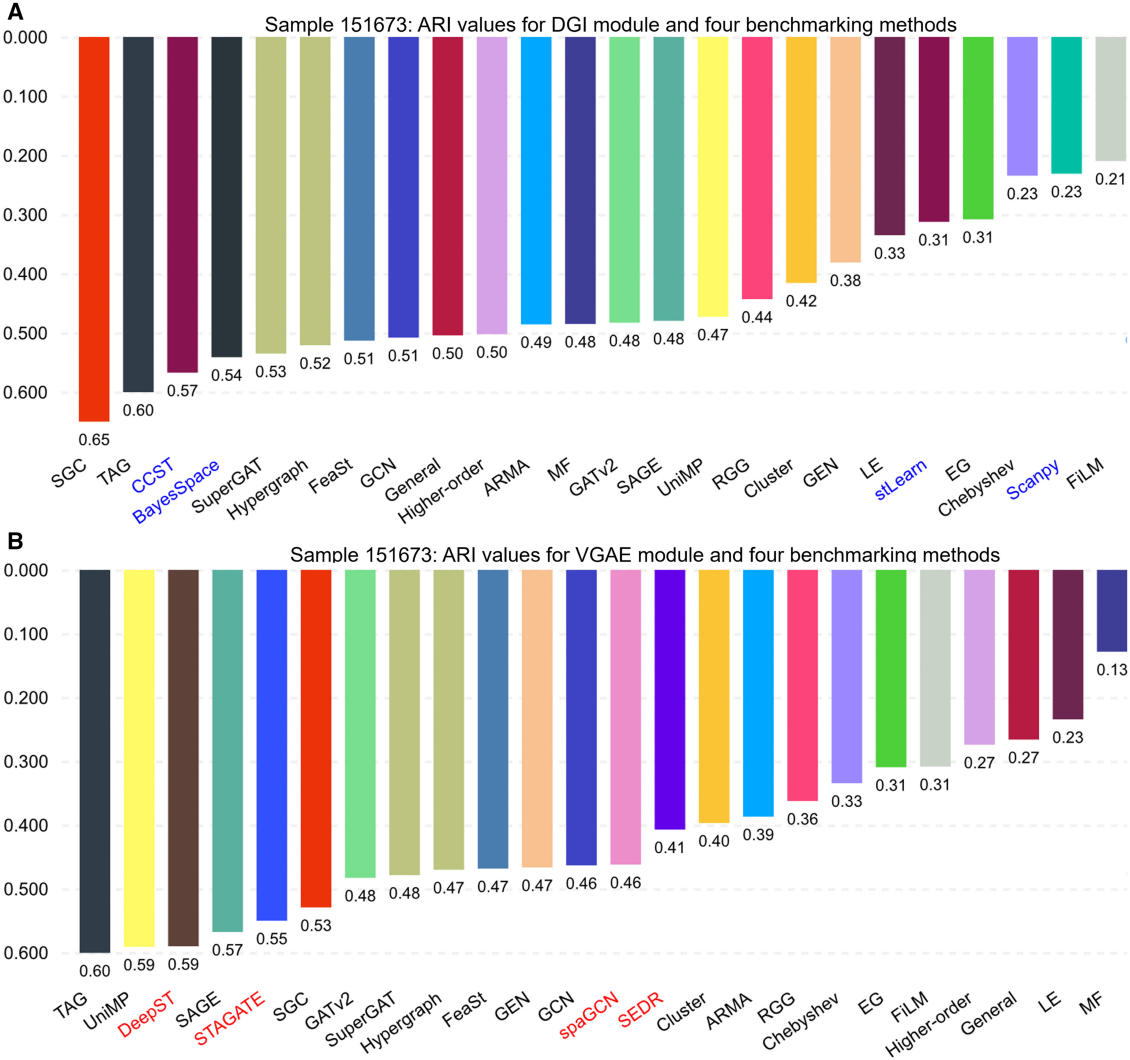
A comparison analysis between GRAPHDeep and eight benchmarking approaches on the Sample 151673 in DLPFC data. (A) Four benchmarking methods are implanted into the DGI module. stLearn and spaGCN are appropriate for this sample because the morphology image is available. (B) DeepST, STAGATE, spaGCN, and SEDR are added to the VGAE module. These four baselines are all related to deep autoencoders. For this sample, the accuracy order of eight benchmarking approaches shift from larger ARI to lower ones is, DeepST, CCST, STAGATE, BayesSpace, spaGCN, SEDR, stLearn, and Scanpy. The top two GNNs in two GDL modules are SGC, TAG, and TAG, UniMP, respectively. This result denotes that GRAPHDeep could build a better spatial clustering framework than the existing algorithms for each spatial omics data.

To encompass the additional baselines (spaGCN and stLearn), we compare GRAPHDeep and the original eight benchmark algorithms on Sample 151673 of human DLPFC dataset with available morphological images. spaGCN is integrated into the VGAE module, and stLearn is integrated into the DGI module (Fig. 6A and B). DGI outperforms the VGAE module for this sample. Although DeepST closely approximates the optimal GNN (TAG) in the VGAE case, the GNNs (SGC and TAG) in DGI outperform DeepST (Fig. 6A and B). STAGATE, BayesSpace, and CCST are middling methods for this sample. ARI values of the remaining baselines do not exceed 0.5. Consequently, the recommended module and GNN for this sample are DGI and SGC. The relevant normalized mutual information scores also demonstrate these findings (Supplementary Fig. S7). These results highlight that the GNNs employed in existing benchmarking methods are suboptimal, underscoring the necessity of GRAPHDeep. The higher ARI values in our experimental results underscore the effectiveness of determining optimal GNNs using GRAPHDeep.

## 4 Discussion

This article unearths multiple insightful conclusions drawn from GRAPHDeep. Firstly, VGAE outperforms the DGI module across most spatial omics data, including 4i, Slide-seqV2, and MERFISH datasets. Secondly, the number of genes or proteins significantly influences spatial clustering algorithms as it dictates feature matrix creation. Similarly, an increase in cells/spots and groups diminishes clustering accuracy and increases computational load. Thirdly, the optimal pairing of GDL module and GNN varies for heterogeneous spatial omics data. Nonetheless, the best GNN can be chosen from a subset of 20 GNNs listed in Supplementary Table S1, such as UniMP, SAGE, Cluster, SuperGAT, FeaSt, GATv2, GCN, and TAG. MF, EG, and LE consistently rank as the poorest GNNs, thus unsuitable for further spatial clustering study. Fourthly, the top time-consuming GNNs across diverse spatial data remain consistent, SuperGAT, GEN, MF, UniMP, and FiLM. When focusing solely on clustering performance, consider the most accurate GNNs. For a trade-off between performance and efficiency, other GNNs may be selected. GRAPHDeep adeptly guides GDL module and GNN selection to meet various optimization goals. Lastly, a comparative analysis between GRAPHDeep and existing spatial clustering algorithms reveals that the GNNs used in the latter are not optimal choices. GRAPHDeep’s selected GNNs prove more efficient and accurate (Figs 5 and 6 and Supplementary Fig. S6).

Quantifying GNNs’ spatial clustering efficacy involves computing their average ranks (Supplementary Fig. S8). This average rank results from summing ranks for each GNN and dividing by four (for four spatial omics data). Comparative analysis of average ranks in DGI and VGAE modules unveils recommended GNNs, such as UniMP, Cluster, SAGE, Chebyshev, GCN, GATv2, FeaSt, and SuperGAT. These GNNs with the lowest average ranks are preferable for future spatial clustering studies. However, specific GNNs, like SGC, Chebyshev, and FiLM, demonstrate distinct performance in these modules, underscoring the significance of GDL module selection for spatial clustering tasks.

In addition to the aforementioned strengths, GRAPHDeep has several limitations. Primarily, incorporating morphological image features into the clustering process could enhance performance, but this option is restricted to specific spatial omics data like 10X Visium and 10X Xenium. Consequently, image-based matrices are alternative inputs in spatial clustering algorithms [e.g. stLearn (Pham *et al.* 2020) and DeepST (Xu *et al.* 2022)], necessitating separate discussion of their learning capabilities. Thus, improvements in clustering performance should be attributed individually to image features, GDL module, or GNN selection. Additionally, GRAPHDeep’s latent embeddings could be enhanced through learning techniques (e.g. iterative and adversarial learning) for better clustering. Integrating traditional methods with learning approaches offers potential to elevate spatial clustering precision. A prospective spatial clustering architecture could formulate the clustering problem as an optimization control issue, optimizing the distance between spots. Moreover, coupling GRAPHDeep with gene imputation techniques could leverage single-cell RNA-sequencing data to enhance gene sequencing depth. The simulation results underscore gene count’s importance in spatial domain identification, making gene imputation a promising strategy for improved clustering performance.

## Supplementary Material

btae023_Supplementary_Data

## Data Availability

The data underlying this article are available in Zenodo Repository, at 10.5281/zenodo.8141084.

## References

[btae023-B1] Berrios DC , Galazka J, Grigorev K et al NASA GeneLab: interfaces for the exploration of space omics data. Nucleic Acids Res 2021;49:D1515–22. 33080015 10.1093/nar/gkaa887 PMC7778922

[btae023-B2] Brody S , Alon U, Yahav E. How attentive are graph attention networks? arXiv, arXiv:2105.14491, 2021, preprint: not peer reviewed.

[btae023-B3] Dong K , Zhang S. Deciphering spatial domains from spatially resolved transcriptomics with an adaptive graph attention auto-encoder. Nat Commun 2022;13:1739. 35365632 10.1038/s41467-022-29439-6 PMC8976049

[btae023-B4] Eng C-HL , Lawson M, Zhu Q et al Transcriptome-scale super-resolved imaging in tissues by RNA seqFISH. Nature 2019;568:235–9. 30911168 10.1038/s41586-019-1049-y PMC6544023

[btae023-B5] Fey M , Lenssen JE. Fast graph representation learning with PyTorch Geometric. arXiv, arXiv:1903.02428, 2019, preprint: not peer reviewed.

[btae023-B6] Xu H , Fu H, Long Y. et al Unsupervised spatially embedded deep representation of spatial transcriptomics. *Genome Med *2024;16 10.1186/s13073-024-01283-x. PMC10790257 38217035

[btae023-B7] Gut G , Herrmann MD, Pelkmans L et al Multiplexed protein maps link subcellular organization to cellular states. Science 2018;361:eaar7042. 30072512 10.1126/science.aar7042

[btae023-B8] Hu J , Li X, Coleman K et al SpaGCN: integrating gene expression, spatial location and histology to identify spatial domains and spatially variable genes by graph convolutional network. Nat Methods 2021;18:1342–51. 34711970 10.1038/s41592-021-01255-8

[btae023-B9] Jackson HW , Fischer JR, Zanotelli VRT et al The single-cell pathology landscape of breast cancer. Nature 2020;578:615–20. 31959985 10.1038/s41586-019-1876-x

[btae023-B10] Keren L , Bosse M, Thompson S et al MIBI-TOF: a multiplexed imaging platform relates cellular phenotypes and tissue structure. Sci Adv 2019;5:eaax5851. 31633026 10.1126/sciadv.aax5851 PMC6785247

[btae023-B11] Kingma D , Welling M. Efficient gradient-based inference through transformations between Bayes nets and neural nets. In: *International Conference on Machine Learning*. Montreal, Quebec, Canada: PMLR, Dec. 8, 2014.

[btae023-B12] Kipf TN , Welling M. Variational graph auto-encoders. arXiv, arXiv:1611.07308, 2016, preprint: not peer reviewed.

[btae023-B13] Kramer B , Castillo JSD, Pelkmans L et al Iterative indirect immunofluorescence imaging (4i) on adherent cells and tissue sections. Bio Protoc 2023;13:e4712. 10.21769/BioProtoc.4712 PMC10336569 37449033

[btae023-B14] Li J , Chen S, Pan X et al Cell clustering for spatial transcriptomics data with graph neural networks. Nat Comput Sci 2022;2:399–408. 38177586 10.1038/s43588-022-00266-5

[btae023-B15] Lu Y , Liu M, Yang J et al Spatial transcriptome profiling by MERFISH reveals fetal liver hematopoietic stem cell niche architecture. Cell Discov 2021;7:47. 34183665 10.1038/s41421-021-00266-1 PMC8238952

[btae023-B16] Moehlin J , Mollet B, Colombo BM et al Inferring biologically relevant molecular tissue substructures by agglomerative clustering of digitized spatial transcriptomes with multilayer. Cell Syst 2021;12:694–705.e3. 34159899 10.1016/j.cels.2021.04.008

[btae023-B17] Palla G , Spitzer H, Klein M et al Squidpy: a scalable framework for spatial omics analysis. Nat Methods 2022;19:171–8. 35102346 10.1038/s41592-021-01358-2 PMC8828470

[btae023-B18] Pardo B , Spangler A, Weber LM et al spatialLIBD: an R/bioconductor package to visualize spatially-resolved transcriptomics data. BMC Genomics 2022;23:434. 35689177 10.1186/s12864-022-08601-w PMC9188087

[btae023-B19] Pham D , Tan X, Xu J et al stLearn: integrating spatial location, tissue morphology and gene expression to find cell types, cell-cell interactions and spatial trajectories within undissociated tissues. bioRxiv, 2020–05, 2020, preprint: not peer reviewed.

[btae023-B20] Prakrithi P , Jain D, Malik PS et al Caution towards spurious off-target signal in 10X visium spatial transcriptomics assay from potential lncRNAs. Brief Bioinform 2023;24:bbad031.

[btae023-B21] Rappez L , Stadler M, Triana S et al SpaceM reveals metabolic states of single cells. Nat Methods 2021;18:799–805. 34226721 10.1038/s41592-021-01198-0 PMC7611214

[btae023-B22] Salas SM , Czarnewski P, Kuemmerle LB et al Optimizing xenium in situ data utility by quality assessment and best practice analysis workflows. bioRxiv, 2023–02, 2023, preprint: not peer reviewed. 10.1038/s41592-025-02617-2 PMC11978515 40082609

[btae023-B23] Stickels RR , Murray E, Kumar P et al Highly sensitive spatial transcriptomics at near-cellular resolution with slide-seqV2. Nat Biotechnol 2021;39:313–9. 33288904 10.1038/s41587-020-0739-1 PMC8606189

[btae023-B24] Traag VA , Waltman L, van Eck NJ et al From Louvain to Leiden: guaranteeing well-connected communities. Sci Rep 2019;9:5233. 30914743 10.1038/s41598-019-41695-z PMC6435756

[btae023-B25] Veličković P , Fedus W, Hamilton WL et al Deep graph infomax. arXiv, arXiv:1809.10341, 2018, preprint: not peer reviewed.

[btae023-B26] Wang Z , Li Y, Li D et al Entropy and gravitation based dynamic radius nearest neighbor classification for imbalanced problem. Knowl Based Syst 2020;193:105474.

[btae023-B27] Wolf FA , Angerer P, Theis FJ et al SCANPY: large-scale single-cell gene expression data analysis. Genome Biol 2018;19:15. 29409532 10.1186/s13059-017-1382-0 PMC5802054

[btae023-B28] Wu Z , Pan S, Chen F et al A comprehensive survey on graph neural networks. IEEE Trans Neural Netw Learn Syst 2021;32:4–24. 32217482 10.1109/TNNLS.2020.2978386

[btae023-B29] Xu C , Jin X, Wei S et al DeepST: identifying spatial domains in spatial transcriptomics by deep learning. Nucleic Acids Res 2022;50:e131. 36250636 10.1093/nar/gkac901 PMC9825193

[btae023-B30] Yang Y , Shi XJ, Liu W et al SC-MEB: spatial clustering with hidden Markov random field using empirical Bayes. Brief Bioinform 2022;23:bbab466. 34849574 10.1093/bib/bbab466 PMC8690176

[btae023-B31] Yuan Z , Zhou Q, Cai L et al SEAM is a spatial single nuclear metabolomics method for dissecting tissue microenvironment. Nat Methods 2021;18:1223–32. 34608315 10.1038/s41592-021-01276-3

[btae023-B32] Zeng Z , Li Y, Li Y et al Statistical and machine learning methods for spatially resolved transcriptomics data analysis. Genome Biol 2022;23:83. 35337374 10.1186/s13059-022-02653-7 PMC8951701

[btae023-B33] Zhao E , Stone MR, Ren X et al Spatial transcriptomics at subspot resolution with BayesSpace. Nat Biotechnol 2021;39:1375–84. 34083791 10.1038/s41587-021-00935-2 PMC8763026

