# Supplementary material for: Assembling spatial clustering framework for heterogeneous spatial transcriptomics data with GRAPHDeep
Source: Bioinformatics. 2024 Jan 18;40(1):btae023. doi: 10.1093/bioinformatics/btae023 (PMC10832355; doi:10.1093/bioinformatics/btae023)
Supplement: btae023_Supplementary_Data [file btae023_supplementary_data.docx]

**Supplementary Materials for**

**Assembling spatial clustering framework for heterogeneous spatial transcriptomics data with GRAPHDeep**

Teng Liu^1,2^, Zhaoyu Fang^3^, Xin Li^1,2^, Lining Zhang^1,2^, Dong-Sheng Cao^4^, Min Li^3^, and Mingzhu Yin^1,2,^*

^1^ Clinical Research Center (CRC), Clinical Pathology Center (CPC), Cancer Early Detection and Treatment Center (CEDTC) and Translational Medicine Research Center (TMRC), Chongqing University Three Gorges Hospital, Chongqing University, Wanzhou, Chongqing, China.

^2^ Chongqing Technical Innovation Center for Quality Evaluation and Identification of Authentic Medicinal Herbs, Chongqing, China.

^3^ Hunan Provincial Key Lab on Bioinformatics, School of Computer Science and Engineering at Central South University, Hunan, China.

^4^ Xiangya School of Pharmaceutical Sciences, Central South University, Changsha, 410003, P.R. China.

* To whom correspondence should be addressed. Tel: [+86-15873141982]; Fax: [0086-731-85202272]; Email: [yinmingzhu2008@126.com]

**1 Supplementary Notes**

**1.1 Pre-processing data and hyper-parameters**

All the pre-processing procedures and computational steps are conducted in Python virtual environment with Pytorch_pyG (Fey and Lenssen, 2019), Squidpy (Palla *et al.*, 2022), and Scanpy (Wolf *et al.*, 2018) toolkits. To generate the feature matrix, the gene expression profiles are normalized and log-transformed first in Scanpy. Then, 3000 highly variable genes are determined and chosen to derive the feature matrix.

If the spatial omics datasets contain fewer than 3000 genes, such as spatial proteomics data, all available genes are employed to construct the feature matrix. To obtain another input (adjacent matrix), the nearest neighbours searching technique in the scikit-learn (Pedregosa *et al.*) toolkit is exploited. The neighbours of each spot are confirmed by either k-neighbours or radius-neighbours mode. For the low-resolution spatial omics data, the radius-neighbours mode is used to guarantee that each spot has approximatively 5 neighbours (Supplementary Fig. S1A). Oppositely, the k-neighbours mode is utilized to make each spot have definitely 5 neighbours (Supplementary Fig. S1B).

The feature and adjacency matrices undergo transformation utilizing graph deep learning modules and graph neural networks, resulting in the creation of latent embeddings. This transformation is implemented in Python using PyTorch_PyG library. The relevant hyperparameters are as follows: input channels totaling 3000 (representing the number of highly variable genes), 128 hidden channels, and 128 output channels. The learning rate is set to 1e-6, the number of epochs to 5000, the weight decay factor to 1e-4, gradient clipping to 5, and the random seed is consistently set to zero across all experiments. The model employs 4 hidden layers, and the activation function outside each layer is the exponential linear unit (ELU). For a fair comparison, all parameters in the variational graph autoencoder (Kipf and Welling, 2016) and deep graph infomax (Veličković *et al.*, 2018) modules are identical.

After producing the latent representations in embedding space, the uniform manifold approximation and projection (UMAP) plots could be described based on the latent embeddings using Scanpy (Wolf *et al.*, 2018). Then, the well-known KMeans clustering method is employed on the latent representations to identify spatial domains and is achieved in the scikit-learn (Pedregosa *et al.*) package. The number of clusters in KMeans is the same as the ground truth. Finally, the compared criterion and downstream figures are further obtained.

The hardware configuration in this paper is Intel (R) Core (TM) i9-12900F CPU @ 2.40 GHz, 64 GB memory, and GeForce RTX 3090Ti GPU. To operate the GNN-based spatial clustering algorithms, the maximum number of cells should be less than 30000. Hence, for some high-resolution spatial omics datasets, the cropped subset of data is used. As this subset keeps the whole genes and clusters, it would not influence the spatial clustering experiments.

**1.2 Spatial clustering metrics**

After generating the prediction labels using KMeans, the adjusted Rand index (ARI) could be applied to estimate the similarity between predicted labels and ground truth. ARI is a frequently-used norm in the evaluation of clustering algorithms. It is also calculated in the scikit-learn toolkit by importing two vectors. Assuming $P=\{P_{1}, P_{2}, \ldots P_{c}\}$ and $G=\{G_{1}, G_{2}, \ldots G_{c}\}$ are the predicted and ground truth labels set, ARI is described as follows:

$$ARI=\frac{\sum_{i,j} \left(\begin{matrix} n_{ij} \\ 2 \end{matrix}\right)-\left[\sum_{i} \left(\begin{matrix} n_{i.} \\ 2 \end{matrix}\right)\sum_{j} \left(\begin{matrix} n_{.j} \\ 2 \end{matrix}\right)\right]/\left(\begin{matrix} n \\ 2 \end{matrix}\right)}{\frac{1}{2}\left[\sum_{i} \left(\begin{matrix} n_{i.} \\ 2 \end{matrix}\right)+\sum_{j} \left(\begin{matrix} n_{.j} \\ 2 \end{matrix}\right)\right]-\left[\sum_{i} \left(\begin{matrix} n_{i.} \\ 2 \end{matrix}\right)\sum_{j} \left(\begin{matrix} n_{.j} \\ 2 \end{matrix}\right)\right]/\left(\begin{matrix} n \\ 2 \end{matrix}\right)}$$

where $c$ is the number of cell types, $n_{i.}$ and $n_{.j}$ denotes the number of spots belonging to $P_{i}$ and $G_{j}$, and $n_{ij}$ implies the number of spots locates in $P_{i}$ and $G_{j}$. A larger ARI means a higher similarity between two classes, and all ARI values are from 0 to 1. As Figure 1E depicted, several statistical characteristics can be computed in Squidpy to express the spatial clustering performance. For example, the centrality scores could depict the degree of aggregation of a single group or two imminent groups. The neighbour enrichment analysis could reflect the proximity relationship of spatial domains. The co-occurrence score is derived according to the conditional probability of two clusters.

**2 Supplementary Figures**

**Fig. S1.** Decision of nearest neighbours to generate the adjacent matrix.

**Fig. S2.** GRAPHDeep’s spatial clustering results on point23 section of MIBI-TOF data.

**Fig. S3.** Consumed time of GRAPHDeep and benchmarking algorithms on Slide-seqV2 data.

**Fig. S4.** A comparison between six benchmarking methods and GRAPHDeep on MERFISH data.

**Fig. S5.** The average rank of 20 GNNs for 4 spatial omics data.


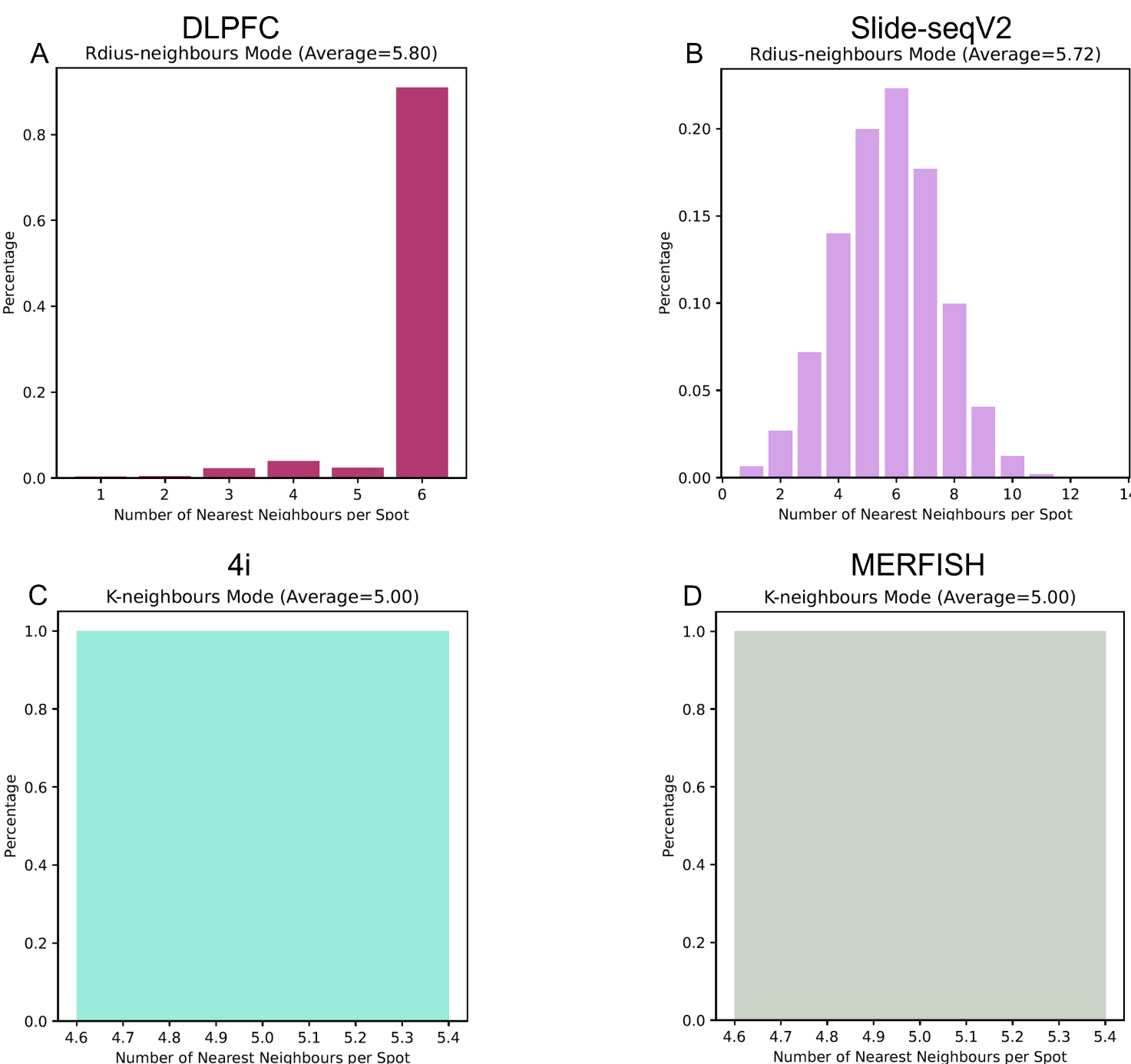


**Fig. S1.** Decision of nearest neighbours to generate the adjacent matrix. **A.** Radius-neighbours mode is employed to search nearest neighbours for low-resolution spatial omics data, such as DLPFC and Slide-seqV2. The spots located in the circle are regarded as the neighbours. The average neighbours of each spot may not be an integer. The radius is determined to keep the average value more than 5 and less than 6. **B.** K-neighbours mode decides the nearest neighbours for high-resolution spatial omics data, such as 4i and MERFISH. For high-resolution spatial data, it is difficult to determine the radius, and thus k-neighbours are treated as the nearest neighbours for each spot. The average neighbours of each spot are integers in this case. K is recognized as 5 for a fair comparison goal.


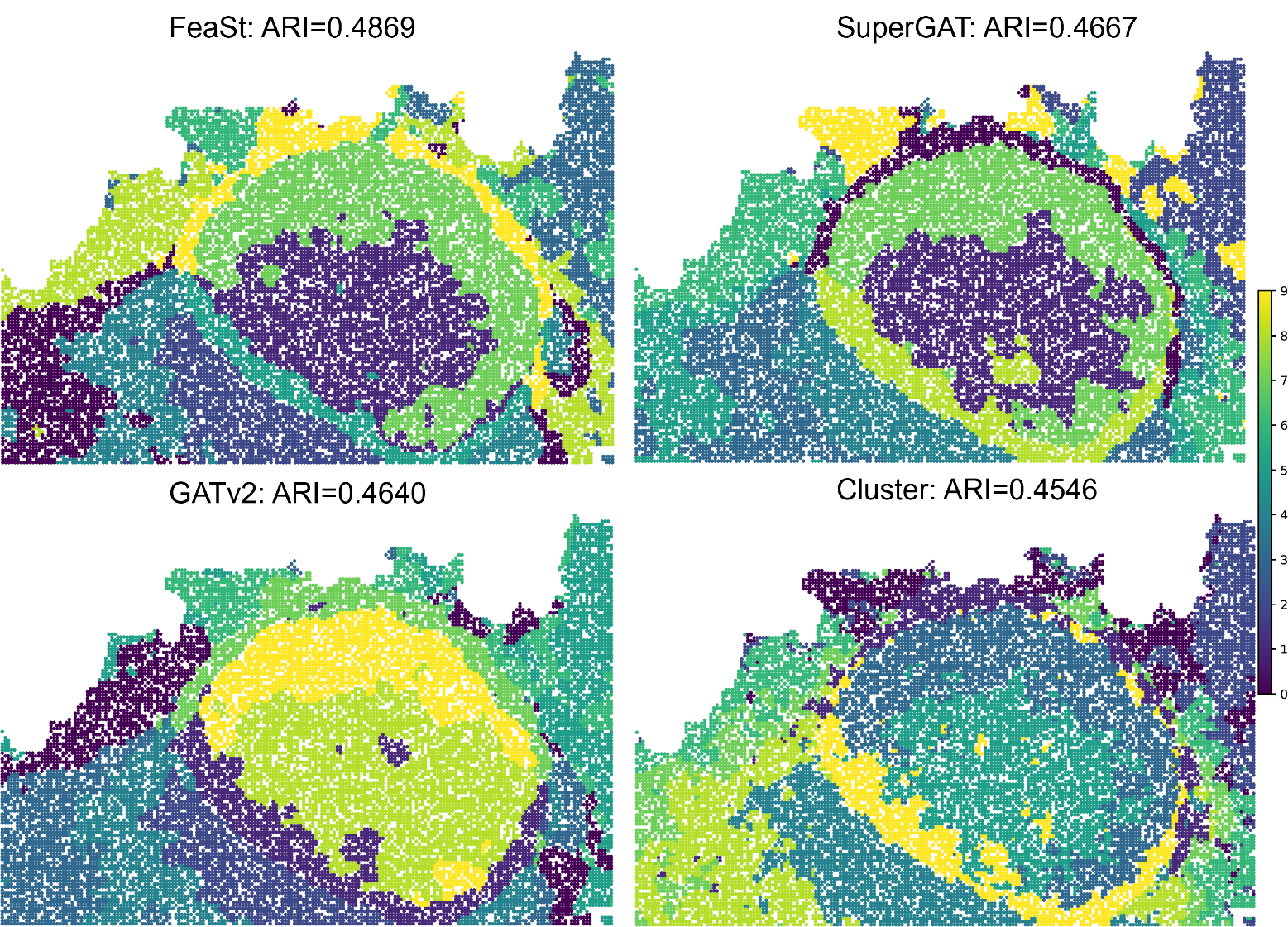


**Fig. S2.** The top four GNNs’ spot distribution in DGI module for 4i spatial protein data.


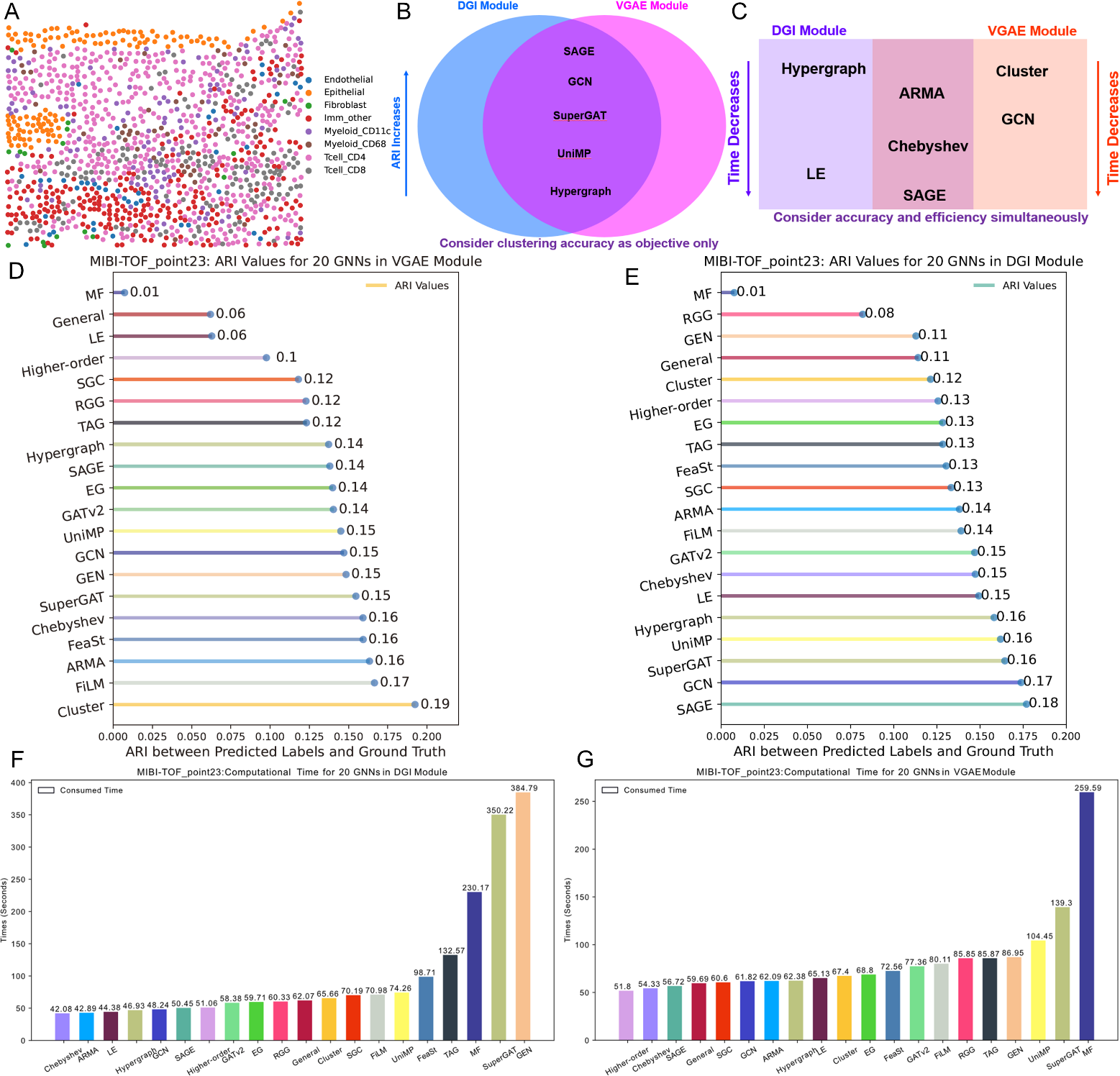


**Fig. S3.** GRAPHDeep’s spatial clustering results on point23 section of MIBI-TOF data. **A.** Ground truth of point23 portion in MIBI-TOF data. This segmentation comprises 1241 cells and 36 proteins in 8 clusters. The spot distribution is tanglesome. **B.** Overlap GNNs between DGI and VGAE modules. The GNNs have the same ARI are assumed to locate at the same rank. **C.** The balance GNNs between VGAE and DGI modules. For each module, the overlaps between top 10 accurate GNNs and the top 10 efficient GNNs are signed first. Here, we list these GNNs and their overlaps. There are three overlap trade-off GNNs. **D.** The ARI distribution in the VGAE module for point23. **E.** The ARI values of 20 GNNs in the DGI module. The lower ARI values are caused by the scarce proteins. The largest ARI are only 0.19 and 0.18. **F.** Computational time with respect to each GNN in the DGI module. **G.** Consumed time of 20 GNNs in the VGAE module.


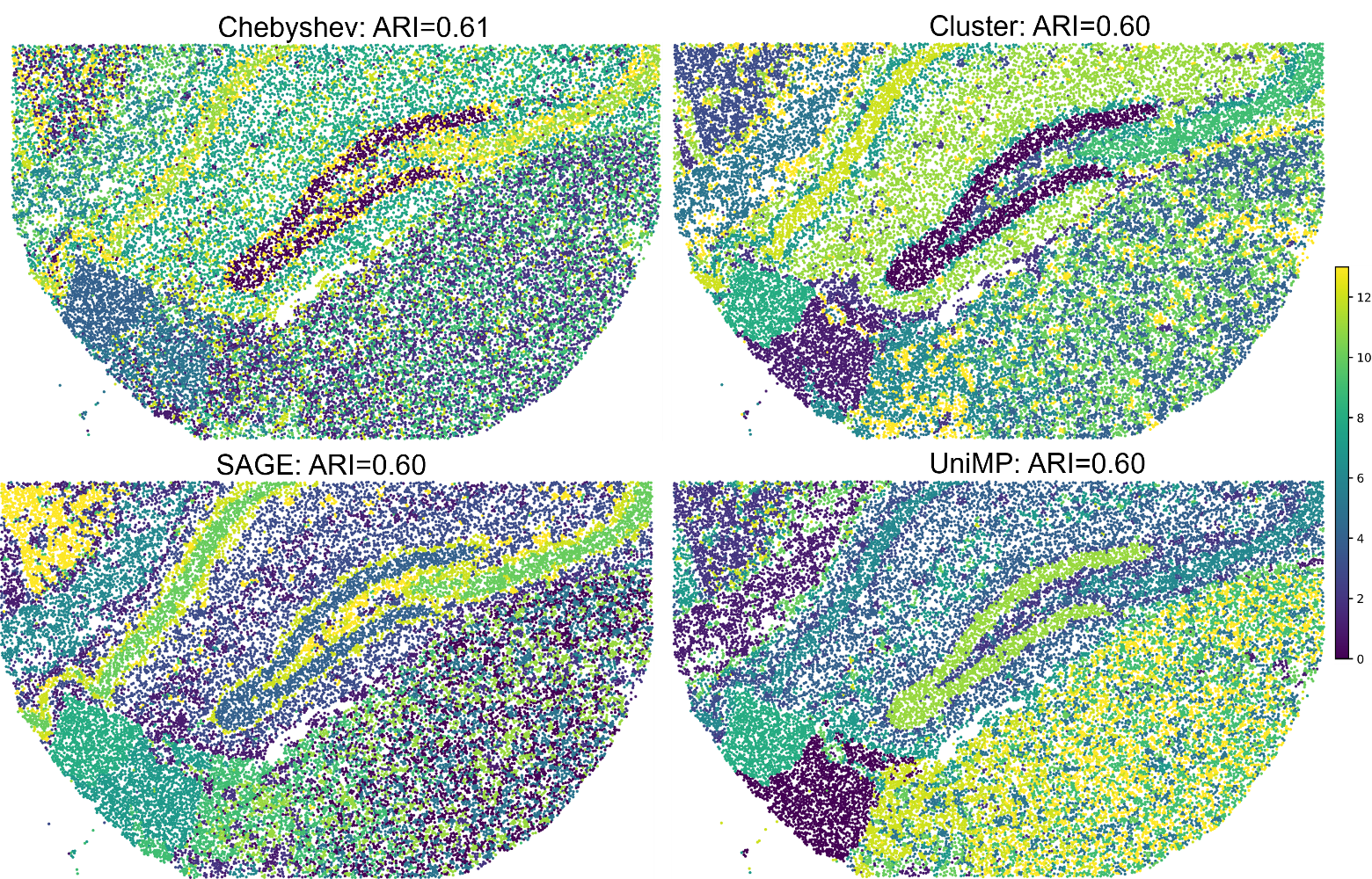


**Fig. S4.** The top four GNNs’ spatial clustering results in VGAE module for Slide-seqV2-based mouse hippocampus data.


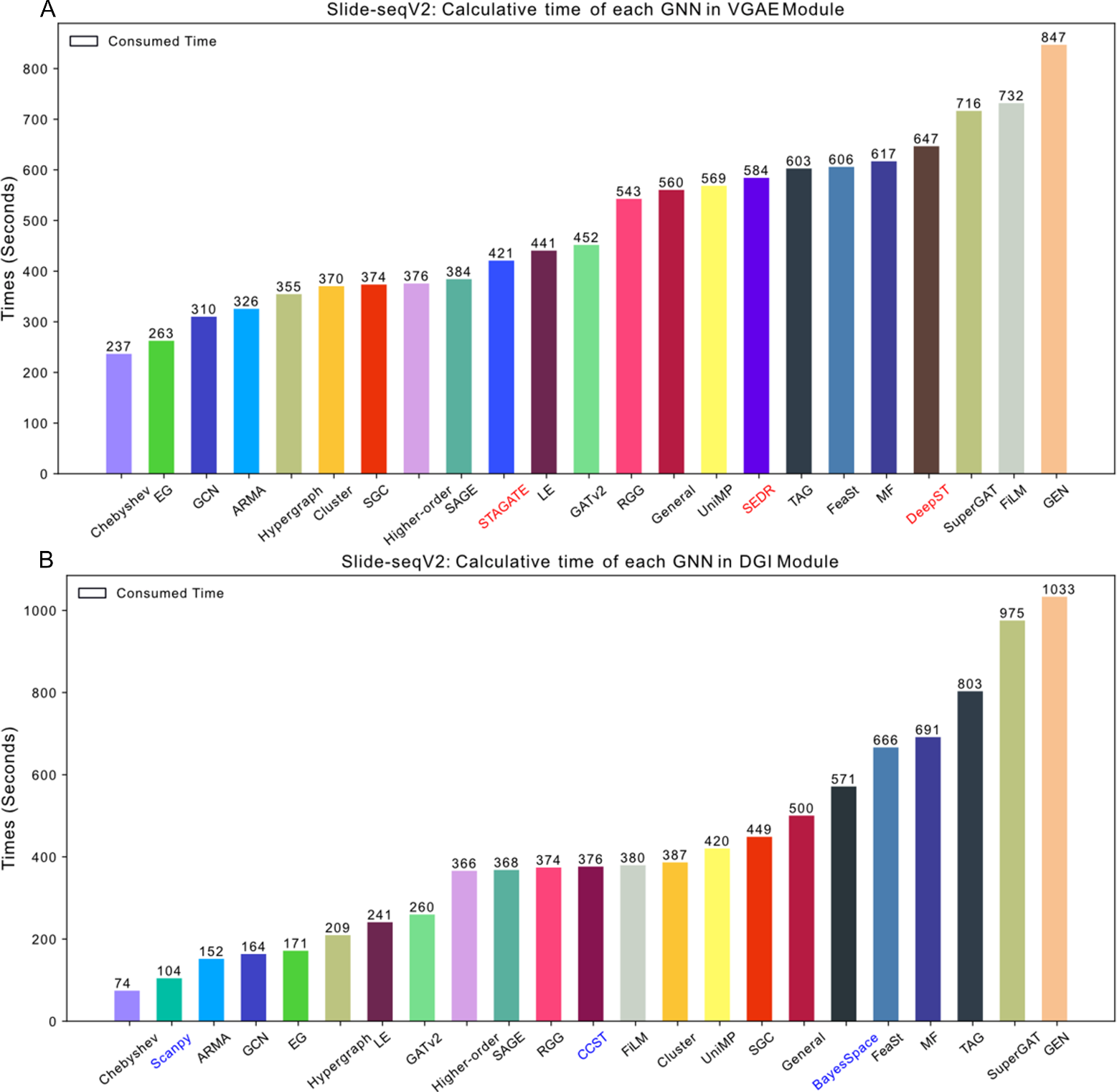


**Fig. S5.** Consumed time of GRAPHDeep and benchmarking algorithms on Slide-seqV2 data. **A.** The elapsed time of 20 GNNs with VGAE and three baseline methods (STAGATE, SEDR, and DeepST). These three benchmarking methods are related to deep autoencoders. The top five time-consuming GNNs are GEN, FiLM, SuperGAT, DeepST, and MF. Chebyshev has the lowest time and highest ARI for this data. **B.** Time distribution of 20 GNNs and three other baseline approaches (CCST, BayesSpace, and Scanpy). Scanpy is a non-spatial clustering method, and its ARI and time are lower. The top five tedious GNNs are similar in two GDL modules, such as GEN, SuperGAT, MF, and FeaSt. These results indicate the elapsed time of each GNN is mainly reflected by its own structure.


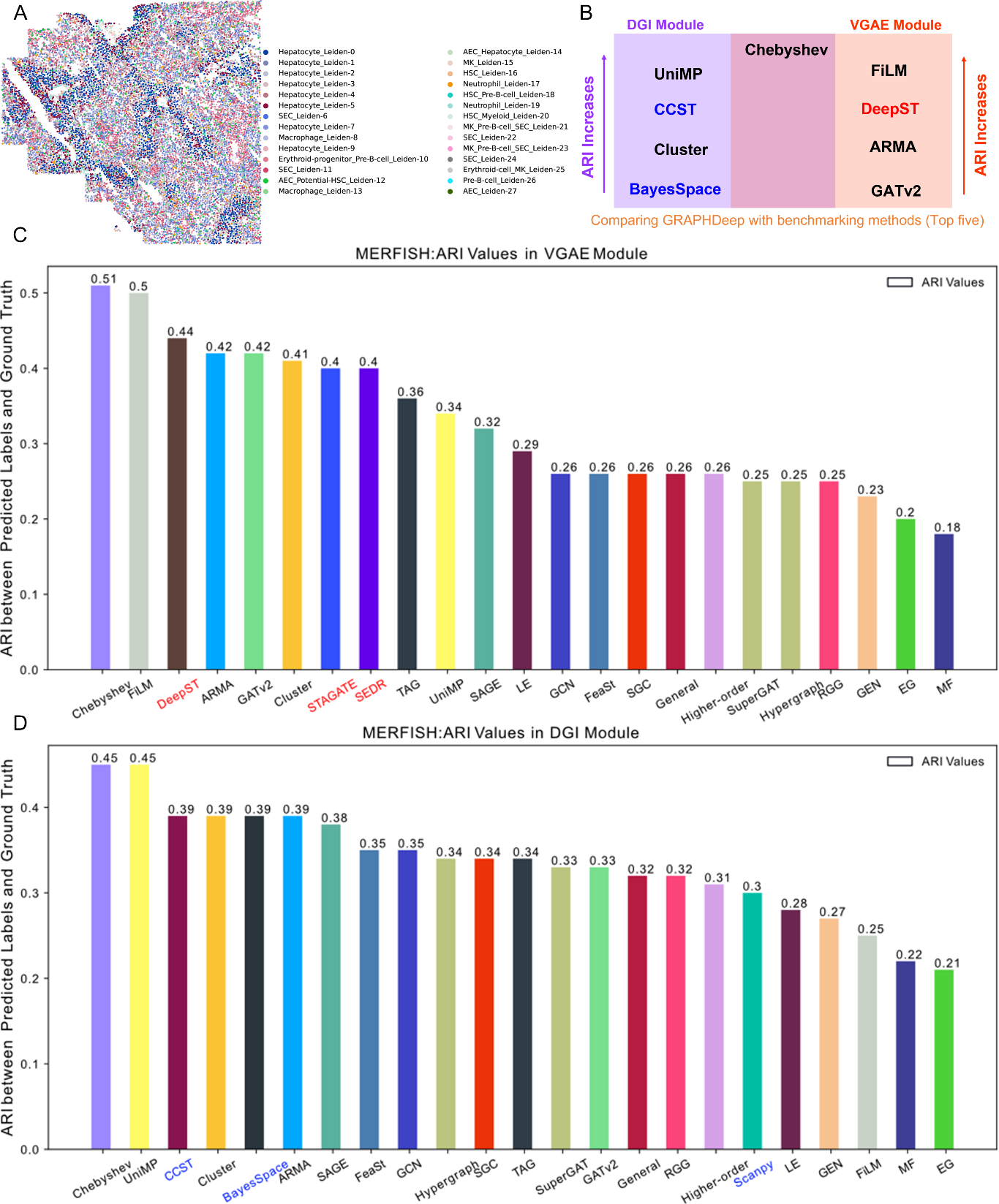


**Fig. S6.** A comparison between six benchmarking methods and GRAPHDeep on MERFISH data. **A.** The ground truth of cropped MERFISH data. This cropped data contains 13806 cells and 347 genes in 28 groups. Compared with Slide-seqV2 data, the number of cells and genes decreases. **B.** Overlap GNNs of two modules in this data. Chebyshev has the highest ARI. DeepST, CCST, and BayesSpace are involved in the top five algorithms. **C.** ARI values in 23 clustering situations, including three benchmarking methods. SEDR equals STAGATE in this condition. These two ARI are also close to GATv2. DeepST is the best choice in six benchmarks. These ARI values are lower than those in Slide-seqV2 data. **D.** ARI values of 20 GNNs and CCST, Scanpy, and BayesSpace. CCST equals to BayesSpace in this data. VGAE performs better than DGI for this data. Chebyshev is the best GNN in VGAE and DGI modules.


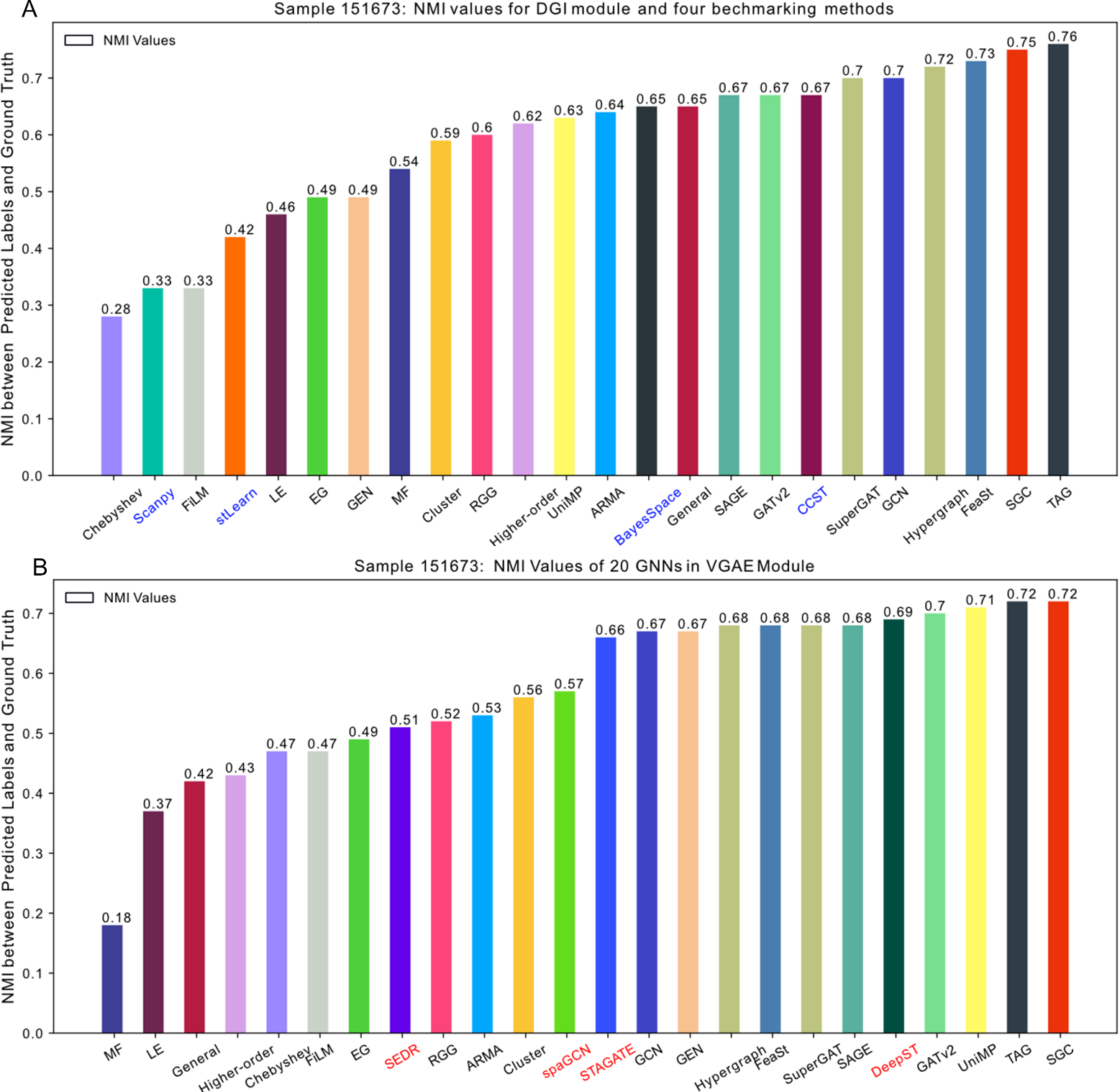


Fig. S7. Normalized mutual information (NMI) scores of GRAPHDeep and eight benchmarking techniques on the sample 151673 in DLPFC data. **A.** Four benchmarking methods are compared with 20 GNNs in DGI module. These methods are Scanpy, stLearn, BayesSpace, and CCST. **B.** NMI values in 20 GNNs in VGAE module are compared with four baselines (SEDR, spaGCN, STAGATE, and DeepST).


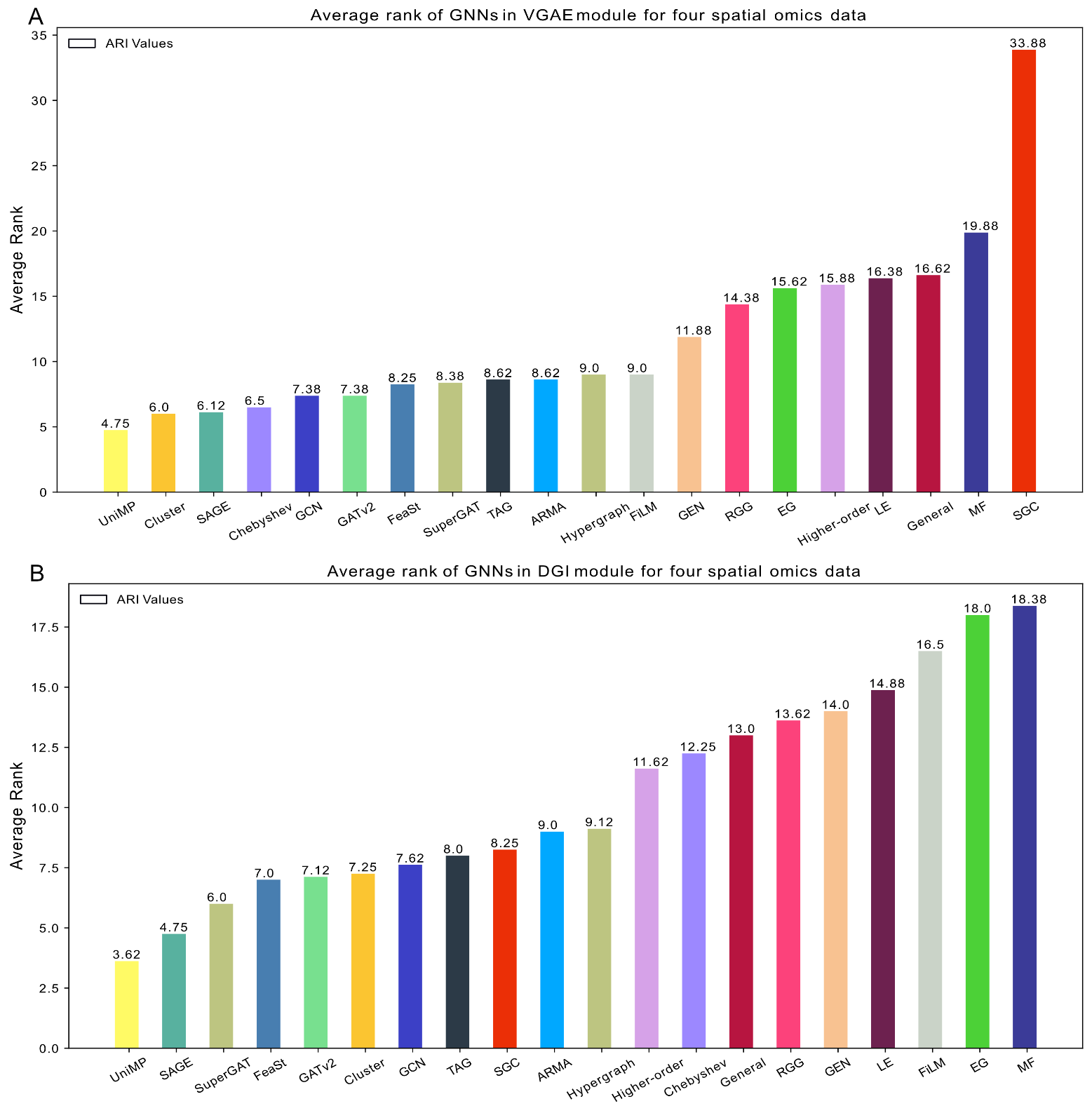


**Fig. S8.** The average rank of 20 GNNs for 4 spatial omics data. **A.** The rank distribution of 20 GNNs in the VGAE module. The average rank equals the total amount of ranks of each GNN times four. A lower rank denotes better performance. The top five lowest ranks in the VGAE module are UniMP, Cluster, SAGE, Chebyshev, and GCN. **B.** The average rank of 20 GNNs in the DGI module. Several GNNs perform dissimilarly in two GDL modules, such as SGC, Chebyshev, and FiLM. The top lowest GNNs are alike in two modules, such as UniMP, SAGE, SuperGAT, GATv2, FeaSt, and Cluster. These GNNs are recommended in further studies.

**3 Supplementary Tables**

**Table S1.** The discussed 20 graph neural networks in GRAPHDeep.

**Table S1.** The discussed 20 graph neural networks in GRAPHDeep.

This table illustrates the abbreviation of each GNN and the relevant released year. The advantages of each GNN are also summarized in this table.

| **GNN Name** | **Years** | **Superiority** |
| --- | --- | --- |
| ARMA (Bianchi *et al.*, 2021) | 2021 | More flexible and robust., better captures the global graph. |
| Chebyshev (Defferrard *et al.*, 2017) | 2017 | Linear computational complexity and constant learning complexity. |
| Cluster (Chiang *et al.*, 2019) | 2019 | Improve memory, calculative efficiency and prediction accuracy. |
| Efficient Graph (Tailor *et al.*, 2022) | 2022 | Better parameter efficiency, latency, and memory consumption. |
| FeaSt (Verma *et al.*, 2018) | 2018 | Learn 3D shape representations from raw input coordinates. |
| FiLM (Brockschmidt, 2020) | 2020 | GNN-FiLM is competitive with or outperforms GNN-MLP. |
| GATv2 (Brody *et al.*, 2022) | 2022 | Higher training accuracy and more robust to edge noise. |
| GEN (Li *et al.*, 2020) | 2020 | Train deep GNN for large scale graphs. |
| General (You *et al.*, 2021) | 2021 | Identify/transfer the best performing architecture quickly. |
| Higher-order (Morris *et al.*, 2019) | 2019 | Work with fine- and coarse-grained structures of a given graph. |
| Hypergraph (Bai *et al.*, 2020) | 2020 | Applications for non-pairwise relationships are observed. |
| LE (Ranjan *et al.*, 2020) | 2020 | Learn functions of local extrema in a subgraph adaptively. |
| MF (Duvenaud *et al.*, 2015) | 2015 | Predictive performance, parsimony, and interpretability. |
| RGG (Bresson and Laurent, 2018) | 2018 | Play an essential role to learn multi-layer architectures. |
| SAGE (Hamilton *et al.*, 2018) | 2018 | Inductive framework to generate embeddings for unseen data. |
| SGC (Wu *et al.*, 2019) | 2019 | Reduce model complexity and redundant computation. |
| SuperGAT (Kim and Oh, 2021) | 2021 | Self-supervised attention using edge information. |
| TAG (Du *et al.*, 2018) | 2018 | Omit approximation, simpler computation, higher accuracy. |
| UniMP (Shi *et al.*, 2021) | 2021 | Integrate GNN and label propagation in semi-supervised learning. |
| GCN (Kipf and Welling, 2017) | 2017 | Most ordinary fast and scalable classification mode. |

Cluster corresponds to ClusterGCNConv in Pytorch_pyG; GEN means GENConv; General corresponds to GeneralConv; Higher-order indicates GraphConv; RGG is ResGatedGraphConv; UniMP corresponds to TransformerConv in Pytorch_pyG.

**Reference**

Bai,S. *et al.* (2020) Hypergraph Convolution and Hypergraph Attention.

Bianchi,F.M. *et al.* (2021) Graph Neural Networks with convolutional ARMA filters. *IEEE Trans. Pattern Anal. Mach. Intell.*, 1–1.

Bresson,X. and Laurent,T. (2018) Residual Gated Graph ConvNets.

Brockschmidt,M. (2020) GNN-FiLM: Graph Neural Networks with Feature-wise Linear Modulation.

Brody,S. *et al.* (2022) How Attentive are Graph Attention Networks?

Chiang,W.-L. *et al.* (2019) Cluster-GCN: An Efficient Algorithm for Training Deep and Large Graph Convolutional Networks. In, *Proceedings of the 25th ACM SIGKDD International Conference on Knowledge Discovery & Data Mining*., pp. 257–266.

Defferrard,M. *et al.* (2017) Convolutional Neural Networks on Graphs with Fast Localized Spectral Filtering.

Du,J. *et al.* (2018) Topology Adaptive Graph Convolutional Networks.

Duvenaud,D. *et al.* (2015) Convolutional Networks on Graphs for Learning Molecular Fingerprints.

Fey,M. and Lenssen,J.E. (2019) Fast Graph Representation Learning with PyTorch Geometric.

Hamilton,W.L. *et al.* (2018) Inductive Representation Learning on Large Graphs.

Kim,D. and Oh,A. (2021) HOW TO FIND YOUR FRIENDLY NEIGHBORHOOD: GRAPH ATTENTION DESIGN WITH SELF-SUPERVISION.

Kipf,T.N. and Welling,M. (2017) Semi-Supervised Classification with Graph Convolutional Networks.

Kipf,T.N. and Welling,M. (2016) Variational Graph Auto-Encoders.

Li,G. *et al.* (2020) DeeperGCN: All You Need to Train Deeper GCNs.

Morris,C. *et al.* (2019) Weisfeiler and Leman Go Neural: Higher-Order Graph Neural Networks. *Proc. AAAI Conf. Artif. Intell.*, **33**, 4602–4609.

Palla,G. *et al.* (2022) Squidpy: a scalable framework for spatial omics analysis. *Nat. Methods*, **19**, 171–178.

Pedregosa,F. *et al.* Scikit-learn: Machine Learning in Python. *Mach. Learn. PYTHON*.

Ranjan,E. *et al.* (2020) ASAP: Adaptive Structure Aware Pooling for Learning Hierarchical Graph Representations.

Shi,Y. *et al.* (2021) Masked Label Prediction: Unified Message Passing Model for Semi-Supervised Classification.

Tailor,S.A. *et al.* (2022) Do We Need Anisotropic Graph Neural Networks?

Veličković,P. *et al.* (2018) Deep Graph Infomax.

Verma,N. *et al.* (2018) FeaStNet: Feature-Steered Graph Convolutions for 3D Shape Analysis.

Wolf,F.A. *et al.* (2018) SCANPY: large-scale single-cell gene expression data analysis. *Genome Biol.*, **19**, 15.

Wu,F. *et al.* (2019) Simplifying Graph Convolutional Networks.

You,J. *et al.* (2021) Design Space for Graph Neural Networks.
